# Connectivity indices of *m*-polar fuzzy network model, with an application to a product manufacturing problem

**DOI:** 10.1007/s10462-022-10360-9

**Published:** 2022-12-21

**Authors:** Muhammad Akram, Saba Siddique, José Carlos R. Alcantud

**Affiliations:** 1 Department of Mathematics, University of the Punjab, New Campus, Lahore, Pakistan; 2 BORDA Research Unit and IME, University of Salamanca, 37007 Salamanca, Spain

**Keywords:** Connectivity index, *m*-Polar fuzzy graphs, Average connectivity index, Special types of vertices.

## Abstract

Connectivity is among the most essential concerns in graph theory and its applications. We consider this issue in a framework that stems from the combination of *m*-polar fuzzy set theory with graphs. We introduce two measurements of connectedness of *m*-polar fuzzy graphs that we call their connectivity and average connectivity indices. Examples are given, and the theoretical performance of these concepts is investigated. Particularly, we are concerned with the effect of deleting a vertex or an edge from an *m*-polar fuzzy graph, on its connectivity and average connectivity indices. We also establish bounding expressions for the connectivity index in complete *m*-polar fuzzy graphs, complete bipartite *m*-polar fuzzy graphs, and wheel *m*-polar fuzzy graphs. Moreover, we introduce some special types of vertices called *m*-polar fuzzy connectivity reducing vertices, *m*-polar fuzzy connectivity enhancing vertices, and *m*-polar fuzzy connectivity neutral vertices. Our theoretical contribution is applied to a product manufacturing problem that takes advantage of multi-polar uncertain information. The justification for our application is systematized using an algorithm. Finally, we compare the proposed method to existing methodologies to demonstrate its feasibility and applicability.

## Introduction

Zadeh (1965) extended the notion of classical subsets of a set to fuzzy sets. In order to indicate uncertainty, he looked at the fundamental concept of degree of membership in a different light. This figure was traditionally used to answer the question whether an element belongs to a subset or not: 0 holds for ‘no’ and 1 means ‘yes’. For the first time his theory allowed an object to belong to a set with a partial degree of membership within [0, 1]. Zadeh’s work (Zadeh 1965) has influenced scientists all across the world. Nowadays, many extensions of his original postulate exist. One of them owes to the view that many issues, from the micro to the macro scale, tend to multi-polarity. As a result, it is no surprise that multi-polarity in information and data collection has gained popularity in many basic and technological disciplines. In neurobiology, for example, multi-polar neurons in the brain collect a lot of information from other neurons. Multi-polar technology may be used to manage large-scale systems in information technology. Bearing this fact in mind, and motivated by the concept of bipolar fuzzy sets (Zhang 1994), Chen et al. (2014) presented the notion of *m*-polar fuzzy (*m*-PF, in short) set as an extension of fuzzy set. The assessment of the membership of an element with *m* different qualities in an *m*-PF set lies in $[0, 1]^m$, and this assessment captures all its separate memberships. This approach is better suited to model a variety of real-world uncertain situations, like the case of data originating with several agents or informational sources. The influence of Zadeh’s paradigm shift extended to graph theory, which is concerned with the relationships among a set of objects under consideration. Its applications demand a precise inspection of technically sound ideas, like those related to connectivity. For example, suppose that we have various routers in a network, then the maximum possible value of the strength of connectedness between two routers is essential to keep its reliability and effectiveness. Also, the only way to confirm the stability of a flow in a piece of the network, or in the entire network, is to measure the average flow in that area. But uncertain relationships abound and they are better captured by fuzzy models. For this reason Kaufmann (1973) set forth the fundamentals of fuzzy graphs. Whereas the strength of connectedness between any two vertices is either 0 or 1 in a graph, it is allowed to be in the range [0, 1] in a fuzzy graph. Connectivity became a decisive concept in fuzzy graph theory too. An issue that demonstrates the importance of connectivity in fuzzy graphs is the fact that total flow disconnection occurs less frequently in physical problems (such as network problems) than flow reduction between pairs of vertices.

Many authors were quick to expand the new theory further. Rosenfeld (1975) investigated fuzzy relations. He studied fuzzy analogues of several basic graph-theoretical notions like cycles and paths, connectedness, bridges and trees. In fact, it was Rosenfeld (1975) who first developed a comprehensive theory of fuzzy graphs, although Yeh and Bang (1975) had independently proposed fuzzy graphs. In addition, they introduced several parameters to assess connectivity in a fuzzy graph, and investigated their applications. Early contributions to the theoretical basis of fuzzy graph theory abound. For example, Mordeson and Nair (2000) presented other operations on fuzzy graphs. Bhattacharya (1987) defined certain concepts of connectivity concerning fuzzy bridges and fuzzy cut-vertices. Bhattacharya and Suraweera (1991) established procedures for the computation of connectivity between pairs of vertices in fuzzy graphs. Banerjee (1991) studied an optimal algorithm for calculating the strength of connectedness in fuzzy graphs, and also Tong and Zheng (1996) developed an algorithm for the calculation of the connectivity matrix of a fuzzy graph. In their analysis of fuzzy graphs, Bhutani and Rosenfeld (2003) established the notions of strong paths and arcs. Mathew and Sunitha (2009) classified distinct types of arcs in fuzzy graphs and developed an arc identification algorithm. A sufficient condition for a node to be a fuzzy cut-node was given in Mathew and Sunitha (2009). Applications and extensions soon appeared as well. Xu (1997) utilized fuzzy graph connectivity parameters to problems involving chemical structures. Binu et al. (2019) examined the connectivity index of fuzzy graphs with application to human trafficking. In the analysis of bipolar fuzzy graphs (Akram 2011), Poulik and Ghorai (2020) introduced certain indices and produced related applications. Recently, Gong et al. (2021) studied domination of bipolar fuzzy graphs. Akram and Waseem (2016) established the concept of *m*-PF graphs in order to study network models with multi-polar, multi-attribute information. Various authors presented additional notions, inclusive of various types of edge *m*-PF graphs (Akram et al. 2017), *m*-PF graph structures (Akram et al. 2016), faces and dual of *m*-PF graphs (Ghorai and Pal 2016a), *m*-PF labeling graphs (Akram and Adeel 2016), fuzzy coloring of *m*-PF graphs (Mahapatra and Pal 2018), and applications of *m*-PF graphs in decision support systems (Akram and Sarwar 2017). The strength of connectedness between vertices has been recently studied in an *m*-PF graph (Mandal et al. 2018; Akram et al. 2021). Mandal et al. (2018) have discussed other fundamental concepts like strong and strongest *m*-PF path, *m*-PF bridge, and *m*-PF forests. Other types of edges in *m*-PF graphs were introduced in Akram et al. (2021). For other concepts, the readers are suggested to Chen (1997), Mahapatra and Pal (2022), Mahapatra et al. (2021), Samanta and Pal (2015), Gao et al. (2022a, b), Habib et al. (2022), and Akram and Nawaz (2022). To summarize, Table 1 presents a brief comparison of works that have used different strategies to develop novel algorithms related to connectivity of graphs.

**Table 1:** Summary of contributions to the study of connectivity of graphs in related literature

Authors and dates	Methodology	Contributions
Mathew and Sunitha (2013)	Cycle connectivity in FGs	1. Proposed the concepts of cycle connectivity of fuzzy trees, fuzzy cycles and complete FGs
		2. Introduced the notions of cyclic cut nodes and cyclic bridges in FGs
Jicy and Mathew (2015)	Connectivity analysis of cyclically balanced FGs	1. Discussed cyclic cut vertices, cyclic bridges and cyclically balanced FGs
		2. Obtained the characterization of cyclically balanced FGs
Ali et al. (2018)	Vertex connectivity of FGs and human trafficking	1. Constructed *t*-connected FGs and average fuzzy vertex connectivity of FGs
		2. Presented the concept of uniformly *t*-connected fuzzy graph
Binu et al. (2019)	Connectivity index of FGs and human trafficking	1. Determined the stability of FGs by the strength of connectedness between each pair of nodes
		2. Introduced two measures namely, connectivity index and average connectivity index of FGs
Poulik and Ghorai (2020)	Certain connectivity indices of bipolar FGs	1. Presented the boundedness of connectivity index of a bipolar FGs
		2. Investigated the changes of connectivity index when a vertex or an edge is removed
		3. Applied the results to increase the popularity of women football league in India and to determine the order of the places to build colleges in a town
Binu et al. (2021)	Connectivity status of fuzzy graphs	1. Adopted connectivity status to build up the status sequence related to FGs
		2. Obtained the results on connectivity status and status sequence of different structures of FGs
Akram et al. (2021)	Menger’s theorem for *m*-PF graphs	1. Classified different types of *m*-PF edges in an *m*PF graph by using the strength of connectedness
		2. Identified different types of *m*-PF edges, including $\alpha$-strong *m*-PF edges, $\beta$-strong *m*-PF edges and $\delta$-weak *m*-PF edges
Naeem et al. (2021)	Connectivity indices of intuitionistic FGs	1. Defined connectivity and average connectivity index for intuitionistic FGs
		2. Described certain kinds of nodes including, connectivity enhancing node, connectivity reducing node and connectivity neutral node for intuitionistic FGs

### Motivation and contributions

Connectivity is the most intuitive attribute to relate with a network. For example, when we have numerous routers on the internet, the maximum possible value of the strength of connectedness between two routers is an essential proxy to guarantee that the network is reliable and effective. Also, the only way to confirm the stability of a flow in a piece of the network or in the entire network is to measure the average flow in that area. The proposed formulation is motivated by the following interests:

- Our main motivation is the lack of a systematic investigation of connectivity in the graph theory generated from multi-polar uncertain information. This is a critical issue that characterizes many real-world decision-making situations.
- Fuzzy and bipolar fuzzy graph models have been successfully employed to manage uncertain information. However both models require further adjustments of membership functions and a great deal of background knowledge.
- Achieving our goal will enable us to enhance the methods described in Binu et al. (2019) and Poulik and Ghorai (2020) which, through their examination of the fuzzy and bipolar fuzzy graph models, have paved the way for decision analysis based on connectivity with fuzzy information.
- In practical applications, the uncertainties in network parameters derived from multi-polar information are far from obvious. Consider for example the case of governments that must decide to implement a smart lockdown during the COVID-19 pandemic. There are many aspects to consider, including the availability of health facilities, testing facilities, public awareness, local rates of transmission, the local community’s reaction to the pandemic, etc. Most of these factors are uncertain in nature. This type of multi-polar uncertain information cannot be correctly handled with the help of fuzzy or bipolar fuzzy graph models. It is thus convenient to pay more attention to connectivity analysis of graphs in the context of *m*-PF graphs.

Motivated by the factors above, our work is devoted to present an extended version of connectivity indices of fuzzy graphs that perform well under multi-polar human assessments. In the current study, we reformulate this method for *m*-PF graphs. The following bullet points encapsulate the innovative contributions of our research work:

- To help studying real-world multi-polar uncertain problems, our research produces two measures of connectedness (connectivity and average connectivity index) for *m*-PF graphs.
- Our study investigates the effects on both connectivity indices of the elimination of a vertex or an edge from an *m*-PF graph.
- We establish bounds of the connectivity index in complete *m*-PF graphs, complete bipartite *m*-PF graphs, wheel *m*-PF graphs. Also, we introduce some special types of vertices, namely, *m*-PF connectivity reducing vertices, *m*-PF connectivity enhancing vertices, and *m*-PF connectivity neutral vertices.
- These new tools are applied to a problem of selection of optimal products to be manufactured by a multinational enterprise.
- A comparison among the fuzzy, bipolar fuzzy, and *m*-PF graph models (through the problem of finding the set of representatives for a youth development council in a university) is also given to prove the flexibility and validity of our new technical contribution.

### Outline of the paper

The content of this paper is organized as follows. Section 2 deals with some basic terminologies and results related to this research work. In Section 3, we define the connectivity index of an *m*-PF graph and use it to prove several theorems on *m*-PF subgraphs. Section 4 deals with bounds for the connectivity index of certain *m*-PF graphs, including complete *m*-PF graphs, complete bipartite *m*-PF graphs and wheel *m*-PF graphs. In Section 5, we discuss the connectivity index of edge-deleted *m*-PF subgraphs of *m*-PF graphs. In Section 6, we define the average connectivity index of an *m*-PF graph and introduce some special types of vertices of *m*-PF graphs using this concept. Section 7 deals with the application of connectivity and average connectivity index of *m*-PF graphs for the selection of products to be manufactured by a company. This section also includes an algorithm to clearly understand the general procedure supporting our application. In Section 8, a comparison of our technique with existing techniques is shown to exhibit the practicality and generality of the method suggested in this paper. Section 9 summarizes the findings, benefits and limitations of our research. The last section 10 concerns conclusions and future directions for research.

## Preliminaries

The concept of *m*-PF set on *W*, a non-empty set, was defined in Chen et al. (2014) as a function $\zeta : W \rightarrow [0, 1]^m$. The collection of all *m*-PF sets on *W* is represented by *m*(*W*). Observe that the set $[0, 1]^m$ can be endowed with a partially ordered set (or poset) structure if we resort to the point-wise order defined by the expression $w \le z$ $\leftrightarrow$ $P_q(w) \le P_q(z)$, where $w, z \in [0, 1]^m$. Here, $P_q : [0, 1]^m \rightarrow [0, 1]$ represents the standard *q*-th projection mapping for every $1 \le q \le m$.

### Definition 1

(Akram and Waseem 2016) Suppose that $\zeta$ is an *m*-PF set on *W*. Then an *m*-PF relation $\sigma$ on $\zeta$ is $\sigma : \zeta \longrightarrow \zeta$, a mapping satisfying that if we write down $\sigma = (P_1 \circ \sigma , P_2 \circ \sigma , \ldots , P_{m} \circ \sigma )$, then for each $w, z \in W$,

$$\begin{aligned} P_q \circ \sigma (wz) \le \inf \{P_q \circ \zeta (w), P_q \circ \zeta (z)\}, \end{aligned}$$

for each $1 \le q \le m$, where $P_q \circ \zeta (w)$ and $P_q \circ \sigma (wz)$ represent the *q*-th membership value of vertex *w* and edge *wz*, respectively. We say that $\sigma$ on *W* is symmetric when $P_q \circ \sigma (wz) = P_q \circ \sigma (zw)$ for each $1 \le q \le m$, where $w, z \in W$.

### Definition 2

(Akram and Waseem 2016) An *m*-PF graph on *W* consists of an ordered pair of mappings ${\mathbb {G}} = (\zeta , \sigma )$, where $\zeta : W \longrightarrow [0, 1]^m$ is an *m*-PF set on *W* and $\sigma : W \times W \longrightarrow [0, 1]^m$ is an *m*-PF relation on *W*, in a such way that for each $wz \in E$,

$$\begin{aligned} P_q \circ \sigma (wz) \le \inf \{P_q \circ \zeta (w), P_q \circ \zeta (z)\}, \end{aligned}$$

for each $1 \le q \le m$ and for all $wz \in W \times W \setminus E$, $\sigma (wz) = \mathbf{0}$. Note that $\mathbf{1} = (1, 1, \ldots , 1)$ and $\mathbf{0} = (0, 0, \ldots , 0)$ are the largest and smallest element of the partial order in $[0, 1]^m$, respectively.

### Definition 3

(Mandal et al. 2018) Consider an *m*-PF graph ${\mathbb {G}} = (\zeta , \sigma )$. An *m*-PF graph $\widetilde{{\mathbb {G}}} = (\widetilde{\zeta }, \widetilde{\sigma })$ is a partial *m*-PF subgraph of ${\mathbb {G}}$ when for each $1 \le q \le m$, it is the case that $P_q \circ \widetilde{\zeta }(w) \le P_q \circ \zeta (w)$ for all $w \in {\widetilde{W}}$ and $P_q \circ \widetilde{\sigma }(wz) \le P_q \circ \sigma (wz)$ for all $wz \in {\widetilde{E}}$. Particularly, a partial *m*-PF subgraph $\widetilde{{\mathbb {G}}} = (\widetilde{\zeta }, \widetilde{\sigma })$ an *m*-PF subgraph of ${\mathbb {G}} = (\zeta , \sigma )$ if for each $1 \le q \le m$, $P_q \circ \widetilde{\zeta }(w) = P_q \circ \zeta (w)$ for all $w \in {\widetilde{W}}$ and $P_q \circ \widetilde{\sigma }(wz) = P_q \circ \sigma (wz)$ for all $wz \in {\widetilde{E}}$. An *m*-PF subgraph $\widetilde{{\mathbb {G}}} = (\widetilde{\zeta }, \widetilde{\sigma })$ spans the *m*-PF graph ${\mathbb {G}} = (\zeta , \sigma )$ if for each $1 \le q \le m$, $P_q \circ \widetilde{\zeta }(w) = P_q \circ \zeta (w)$ for all $w \in W$, i.e., $W = {\widetilde{W}}$ and $\zeta (w) = \widetilde{\zeta }(w)$ for all $w \in W = {\widetilde{W}}$.

### Definition 4

(Akram and Waseem 2016) Consider an *m*-PF graph ${\mathbb {G}} = (\zeta , \sigma )$. An *m*-PF path *P* in ${\mathbb {G}}$ consists of a sequence of different vertices $w = w_1, w_2, w_3, \ldots , w_n = z$ such that there exists at least one $1 \le q \le m$ for each $1 \le i \le n - 1$ satisfying $P_q \circ \sigma (w_i w_{i+1}) >0$. An *m*-PF path between two vertices $w_i$ and $w_k$ is said to be an *m*-PF cycle *C* (Akram et al. 2021) if $w_i = w_k$ and $n \ge 3$. The strength of an *m*-PF path *P* is described as $S(P) = (\inf \limits _{1 \le i< k \le n} P_1 \circ \sigma (w_iw_{k}), \inf \limits _{1 \le i< k \le n} P_2 \circ \sigma (w_iw_{k}), \ldots , \inf \limits _{1 \le i < k \le n} P_m \circ \sigma (w_iw_{k}))$. The strength of connectedness between two vertices $w_i$ and $w_k$ ($CONN_{{\mathbb {G}}}(w_i, w_k)$) is the maximum of the strengths of all *m*-PF paths between $w_i$ and $w_k$. Mathematically, it is defined as $CONN_{{\mathbb {G}}}(w_i, w_k) = ((P_1 \circ \sigma (w_iw_{k}))^{\infty }, (P_2 \circ \sigma (w_iw_{k}))^{\infty }, \ldots , (P_m \circ \sigma (w_iw_{k}))^{\infty })$, where $(P_q \circ \sigma (w_iw_{k}))^{\infty } = \max \{\inf \limits _{1 \le i < k \le n} P_q \circ \sigma (w_iw_{k})\}$. An *m*-PF path is defined to be a strongest *m*-PF path if $S(P) = CONN_{{\mathbb {G}}}(w_i, w_k)$. An *m*-PF graph ${\mathbb {G}} = (\zeta , \sigma )$ is connected if there exists an *m*-PF path between each pair of vertices, i.e., $(P_q \circ \sigma (w_iw_{k}))^{\infty } > 0$ for at least one *q*.

In the rest of this paper, we suppose that the *m*-PF graph ${\mathbb {G}}$ is connected.

### Definition 5

(Mandal et al. 2018) A connected *m*-PF graph ${\mathbb {G}} = (\zeta , \sigma )$ is an *m*-PF tree if it has an *m*-PF spanning subgraph $\widetilde{{\mathbb {G}}} = (\widetilde{\zeta }, \widetilde{\sigma })$ which is a tree and for each edge *wz* not in $\widetilde{{\mathbb {G}}}$ there exists an *m*-PF path from *w* to *z* in $\widetilde{{\mathbb {G}}}$ whose strength is greater than the membership value of edge *wz* in ${\mathbb {G}}$, i.e., $P_q \circ CONN_{\widetilde{{\mathbb {G}}}}(w, z) > P_q \circ \sigma (wz)$ for each $1 \le q \le m$.

Note that $\widetilde{{\mathbb {G}}}$ is a tree such that it contains all the vertices of ${\mathbb {G}}$, therefore, it is in fact a spanning tree of ${\mathbb {G}}$. Yet more, ${\mathbb {G}}$ has no other maximum spanning tree.

### Definition 6

(Akram and Waseem 2016) An *m*-PF graph ${\mathbb {G}} = (\zeta , \sigma )$ is a strong *m*-PF graph when for all $wz \in E$, $P_q \circ \sigma (wz) = \inf \{P_q \circ \zeta (w), P_q \circ \zeta (z)\}$ for each $1 \le q \le m$. We say that the *m*-PF graph ${\mathbb {G}} = (\zeta , \sigma )$ is complete when for all $w, z \in W$, $P_q \circ \sigma (wz) = \inf \{P_q \circ \zeta (w), P_q \circ \zeta (z)\}$ for each $1 \le q \le m$.

### Definition 7

(Akram et al. 2021) An *m*-PF graph ${\mathbb {G}} = (\zeta , \sigma )$ is a bipartite *m*-PF graph if $W = W_1 \cup W_2$ ($W_1 \cap W_2 = \emptyset$) such that (i). $P_q \circ \sigma (wz) = 0$ for each $1 \le q \le m$, if $w, z \in W_1$ or $w, z \in W_2$, (ii). $P_q \circ \sigma (wz) > 0$ for at least one *q*, if $w \in W_1$ and $z \in W_2$ or $w \in W_2$ and $z \in W_1$.

### Definition 8

(Akram et al. 2021) We say that the *m*-PF graph ${\mathbb {G}} = (\zeta , \sigma )$ is a complete bipartite *m*-PF graph when $W = W_1 \cup W_2$ ($W_1 \cap W_2 = \emptyset$) such that (i). $P_q \circ \sigma (wz) = 0$ for each $1 \le q \le m$, if $w, z \in W_1$ or $w, z \in W_2$, (ii). $P_q \circ \sigma (wz) = \inf \{P_q \circ \zeta (w), P_q \circ \zeta (z)\}$ for each $1 \le q \le m$, if $w \in W_1$ and $z \in W_2$ or $w \in W_2$ and $z \in W_1$.

### Definition 9

(Akram et al. 2021) Suppose that ${\mathbb {G}} = (\zeta , \sigma )$ is an *m*-PF graph. We say that the edge $wz \in E$ is a strong *m*-PF edge of ${\mathbb {G}}$ when $P_q \circ \sigma (wz) \ge P_q \circ CONN_{{\mathbb {G}} \setminus \{wz\}}(w, z)$, for each $1 \le q \le m$. The vertices *w* and *z*, incident to a strong *m*-PF edge, are strong *m*-PF neighbors when $P_q \circ \sigma (wz) > 0$ for each $1 \le q \le m$. An *m*-PF path *P* between *w* and *z* is strong when all edges contributing to the path are strong.

A strong *m*-PF edge $wz \in E$ is called an $\alpha$-strong *m*-PF edge ($\alpha$-edge) if for each $1 \le q \le m$, $P_q \circ \sigma (wz) > P_q \circ CONN_{{\mathbb {G}} \setminus \{wz\}}(w, z)$. A strong *m*-PF edge $wz \in E$ is called a $\beta$-strong *m*-PF edge ($\beta$-edge) if for each $1 \le q \le m$, $P_q \circ \sigma (wz) = P_q \circ CONN_{{\mathbb {G}} \setminus \{wz\}}(w, z)$. An edge $wz \in E$ is a weak *m*-PF edge of ${\mathbb {G}}$ if it is not strong *m*-PF edge of ${\mathbb {G}}$. A weak *m*-PF edge *wz* is $\delta$-weak (or a $\delta$-edge) if for each $1 \le q \le m$, $P_q \circ \sigma (wz) < P_q \circ CONN_{{\mathbb {G}} \setminus \{wz\}}(w, z)$. Otherwise, we say that it is a mixed *m*-PF edge ($\textit{M}$-edge) of ${\mathbb {G}}$.

### Definition 10

(Akram et al. 2021) Let ${\mathbb {G}} = (\zeta , \sigma )$ be an *m*-PF graph. We say that the edge $wz \in E$ is an *m*-PF bridge of ${\mathbb {G}}$ when we get a partial *m*-PF subgraph $\widetilde{{\mathbb {G}}} = {\mathbb {G}} \setminus \{wz\}$ by replacing $\sigma (wz) = \mathbf{0} = \widetilde{\sigma }(wz)$ in which for each $1 \le q \le m$, either $P_q \circ CONN_{{\mathbb {G}} \setminus \{wz\}}(u, v) = 0$ or $P_q \circ CONN_{{\mathbb {G}} \setminus \{wz\}}(u, v) < P_q \circ CONN_{{\mathbb {G}}}(u, v)$ for some pair of vertices *u* and *v* of ${\mathbb {G}} \setminus \{wz\}$.

### Definition 11

(Akram et al. 2021) Suppose that ${\mathbb {G}} = (\zeta , \sigma )$ is an *m*-PF graph. We say that the edge $ab \in E$ is a weakest *m*-PF edge of ${\mathbb {G}}$, when for each $1 \le q \le m$, $P_q \circ \sigma (ab) < P_q \circ \sigma (wz)$ for any edge $wz \in E$ other than *ab*. And the edge $cd \in E$ is a strongest *m*-PF edge of ${\mathbb {G}}$, when for each $1 \le q \le m$, $P_q \circ \sigma (cd) > P_q \circ \sigma (wz)$ for any edge $wz \in E$ other than *cd*.

### Definition 12

(Ghorai and Pal 2016b) Let ${\mathbb {G}}_1 = (\zeta _1, \sigma _1)$ and ${\mathbb {G}}_2 = (\zeta _2, \sigma _2)$ be two *m*-PF graphs of the crisp graphs ${\mathbb {G}}^{\star }_1 = (W_1, E_1)$ and ${\mathbb {G}}^{\star }_2 = (W_2, E_2)$, respectively.

1. If there exists a mapping $\xi : W_1 \longrightarrow W_2$ such that for each $1 \le q \le m$, $P_q \circ \zeta _1(w) \le P_q \circ \zeta _2(\xi (w))$ for all $w \in W_1$ and $P_q \circ \sigma _1(wz) \le P_q \circ \sigma _2(\xi (w)\xi (z))$ for all $wz \in E_1$, then the mapping $\xi : W_1 \longrightarrow W_2$ is called a homomorphism between ${\mathbb {G}}_1$ and ${\mathbb {G}}_2$.
2. If there is a bijective map $\xi : W_1 \longrightarrow W_2$ with the property that for each $1 \le q \le m$, $P_q \circ \zeta _1(w) = P_q \circ \zeta _2(\xi (w))$ for all $w \in W_1$ and $P_q \circ \sigma _1(wz) = P_q \circ \sigma _2(\xi (w)\xi (z))$ for all $wz \in E_1$, then the mapping $\xi : W_1 \longrightarrow W_2$ is called an isomorphism between ${\mathbb {G}}_1$ and ${\mathbb {G}}_2$.

## Connectivity index of an *m*-PF graph

Connectivity is the most intuitive attribute to relate with a network. For instance, when we have many routers in a network, the maximum possible value of the strength of connectedness between two routers is essential to keep the network more reliable and effective. In this section, we first define the connectivity index of an *m*-PF graph ${\mathbb {G}} = (\zeta , \sigma )$, we then prove several theorems on *m*-PF subgraphs using the connectivity index of ${\mathbb {G}}$. The definition of connectivity index of an *m*-PF graph ${\mathbb {G}} = (\zeta , \sigma )$ is given below:

### Definition 13

Let ${\mathbb {G}} = (\zeta , \sigma )$ be an *m*-PF graph. The connectivity index of ${\mathbb {G}}$ is denoted by $CI({\mathbb {G}})$ and is defined as $CI({\mathbb {G}}) = (P_1 \circ CI({\mathbb {G}}), P_2 \circ CI({\mathbb {G}}), \ldots , P_m \circ CI({\mathbb {G}}))$, where $P_q \circ CI({\mathbb {G}}) = \sum _{w, z \in W} P_q \circ \zeta (w) P_q \circ \zeta (z) P_q \circ CONN_G(w, z)$ for each $1 \le q \le m$.

Note that, unless otherwise mentioned, the considered examples of *m*-PF graphs in the following sections will have for each $1 \le q \le m$, $P_q \circ \zeta (w) = 1$ for all $w \in W$.

### Example 1

**Fig. 1:**
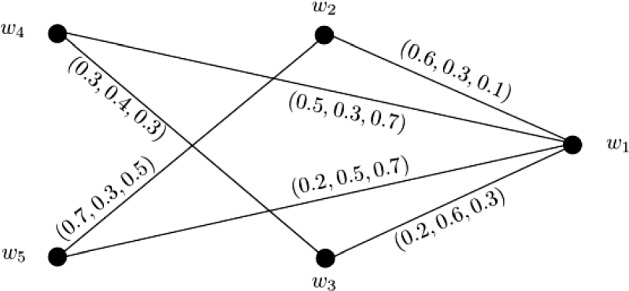
3-PF graph

Let $W = \{w_1, w_2, w_3, w_4, w_5\}$ be a vertex set and $E = \{w_1w_2, w_1w_3, w_1w_4, w_1w_5, w_2w_5, w_3w_4\}$ be the set of edges. The corresponding 3-PF graph ${\mathbb {G}} = (\zeta , \sigma )$ is given in Fig. 1 with $\zeta (w_i) = (1, 1, 1)$ for all $i = 1, 2, \ldots , 5$. Consider two vertices $w_2$ and $w_4$. The 3-PF paths from $w_2$ to $w_4$ with their corresponding strengths are given below:

$$\blacktriangleright$$
$$P^1$$: $$w_2 - w_1 - w_4$$ $$S(P^1) = (0.5, 0.3, 0.1)$$,
$$\blacktriangleright$$
$$P^2$$: $$w_2 - w_1 - w_3 - w_4$$ $$S(P^2) = (0.2, 0.3, 0.1)$$,
$$\blacktriangleright$$
$$P^3$$: $$w_2 - w_5 - w_1 - w_4$$ $$S(P^3) = (0.2, 0.3, 0.5)$$,
$$\blacktriangleright$$
$$P^4$$: $$w_2 - w_5 - w_1 - w_3 - w_4$$ $$S(P^4) = (0.2, 0.3, 0.3)$$.

The strength of connectedness between $w_2$ and $w_4$ is $CONN_{{\mathbb {G}}}(w_2, w_4) = (0.5, 0.3, 0.5)$. Similarly, the strength of connectedness between all pairs of vertices of ${\mathbb {G}}$ are calculated in the following connectivity matrix (1). Since, $\zeta (w_i) = (1, 1, 1)$ for all $i = 1, 2, \ldots , 5$ and connectivity matrix of ${\mathbb {G}}$ is symmetric, therefore, the $CI({\mathbb {G}})$ can be obtained by taking the summation of all entries in the lower or upper triangular entries of this connectivity matrix. After calculations, we have $CI({\mathbb {G}}) = (4.6, 3.3, 4.8)$.

1 $$\begin{aligned}&\left[ \begin{array}{ccccc} (0.0, 0.0, 0.0) &{} (0.6, 0.3, 0.5) &{} (0.3, 0.6, 0.3) &{} (0.5, 0.4, 0.7) &{} (0.6, 0.5, 0.7)\\ (0.6, 0.3, 0.5) &{} (0.0, 0.0, 0.0) &{} (0.3, 0.3, 0.3) &{} (0.5, 0.3, 0.5) &{} (0.7, 0.3, 0.5)\\ (0.3, 0.6, 0.3) &{} (0.3, 0.3, 0.3) &{} (0.0, 0.0, 0.0) &{} (0.3, 0.4, 0.3) &{} (0.3, 0.3, 0.3)\\ (0.5, 0.4, 0.7) &{} (0.5, 0.3, 0.5) &{} (0.3, 0.4, 0.3) &{} (0.0, 0.0, 0.0) &{} (0.5, 0.3, 0.7)\\ (0.6, 0.5, 0.7) &{} (0.7, 0.3, 0.5) &{} (0.3, 0.3, 0.3) &{} (0.5, 0.3, 0.7) &{} (0.0, 0.0, 0.0)\\ \end{array} \right] \end{aligned}$$

In general, *m*-PF subgraphs will have fewer connections than the *m*-PF graph and their connectivity indices will never be higher. As a result, we arrive to the following proposition.

### Proposition 1

Let ${\mathbb {G}} = (\zeta , \sigma )$ be an *m*-PF graph and $\widetilde{{\mathbb {G}}} = (\widetilde{\zeta }, \widetilde{\sigma })$ be a partial *m*-PF subgraph of ${\mathbb {G}}$. Then for each $1 \le q \le m$, $P_q \circ CI(\widetilde{{\mathbb {G}}}) \le P_q \circ CI({\mathbb {G}})$, that is, $CI(\widetilde{{\mathbb {G}}})$ will be less than or equal to $CI({\mathbb {G}})$.

### Proof

Let ${\mathbb {G}} = (\zeta , \sigma )$ be an *m*-PF graph and $\widetilde{{\mathbb {G}}} = (\widetilde{\zeta }, \widetilde{\sigma })$ be a partial *m*-PF subgraph of ${\mathbb {G}}$. Then it is clear that for each $1 \le q \le m$, $P_q \circ \widetilde{\zeta }(w) \le P_q \circ \zeta (w)$ and $P_q \circ \widetilde{\sigma }(wz) \le P_q \circ \sigma (wz)$ for all $w \in {\widetilde{W}}$ and $wz \in {\widetilde{E}}$. This implies that for each $1 \le q \le m$, $P_q \circ CONN_{\widetilde{{\mathbb {G}}}}(w, z) \le P_q \circ CONN_{{\mathbb {G}}}(wz)$ for all $w, z \in {\widetilde{W}}$. Since, for each $1 \le q \le m$, $P_q \circ \widetilde{\zeta }(a) \ge 0$ and $P_q \circ \zeta (b) \ge 0$ for all $a \in {\widetilde{W}}$ and for all $b \in W$, respectively. Therefore, for each $1 \le q \le m$, $P_q \circ \widetilde{\zeta }(w)P_q \circ \widetilde{\zeta }(z)P_q \circ CONN_{\widetilde{{\mathbb {G}}}}(w, z) \le P_q \circ \zeta (w)P_q \circ \zeta (z)P_q \circ CONN_{{\mathbb {G}}}(w, z)$. This means that for each $1 \le q \le m$, $\sum _{w, z \in {\widetilde{W}}} P_q \circ \widetilde{\zeta }(w)P_q \circ \widetilde{\zeta }(z)P_q \circ CONN_{\widetilde{{\mathbb {G}}}}(w, z) \le \sum _{w, z \in W} P_q \circ \zeta (w)P_q \circ \zeta (z)P_q \circ CONN_{{\mathbb {G}}}(w, z)$. Thus, for each $1 \le q \le m$, $P_q \circ CI(\widetilde{{\mathbb {G}}}) \le P_q \circ CI({\mathbb {G}})$. This shows that $CI(\widetilde{{\mathbb {G}}})$ can never exceed $CI({\mathbb {G}})$. $\square$

Note that Proposition 1 holds true for *m*-PF subgraphs of an *m*-PF graph ${\mathbb {G}}$ as every *m*-PF subgraph of ${\mathbb {G}}$ is a partial *m*-PF subgraph. Consider the following example.

### Example 2

**Fig. 2:**
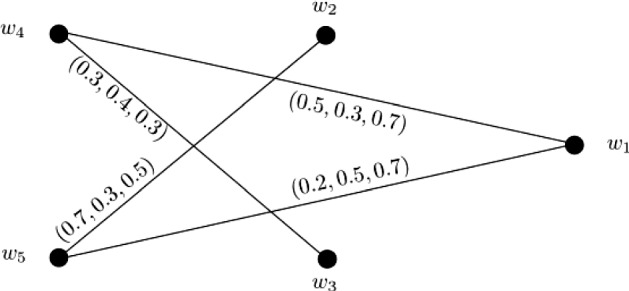
3-PF graph

Let ${\widetilde{W}} = \{w_1, w_2, w_3, w_4, w_5\}$ be a vertex set and ${\widetilde{E}} = \{w_1w_4, w_1w_5, w_2w_5, w_3w_4\}$ be the set of edges. The 3-PF graph $\widetilde{{\mathbb {G}}} = (\widetilde{\zeta }, \widetilde{\sigma })$ associated with this data is shown in Fig. 2 with $\widetilde{\zeta }(w_i) = (1, 1, 1)$ for all $i = 1, 2, \ldots , 5$. Clearly, it is a 3-PF subgraph of the 3-PF graph ${\mathbb {G}} = (\zeta , \sigma )$ in Example 1 (Fig. 1). The strength of connectedness between all pairs of vertices of $\widetilde{{\mathbb {G}}}$ are calculated in the connectivity matrix (2). After calculations, we have $CI(\widetilde{{\mathbb {G}}}) = (3.0, 3.3, 4.8)$.

2 $$\begin{aligned}&\left[ \begin{array}{ccccc} (0.0, 0.0, 0.0) &{} (0.2, 0.3, 0.5) &{} (0.3, 0.3, 0.3) &{} (0.5, 0.3, 0.7) &{} (0.2, 0.5, 0.7)\\ (0.2, 0.3, 0.5) &{} (0.0, 0.0, 0.0) &{} (0.2 0.3, 0.3) &{} (0.2, 0.3, 0.5) &{} (0.7, 0.3, 0.5)\\ (0.3, 0.3, 0.3) &{} (0.2 0.3, 0.3) &{} (0.0, 0.0, 0.0) &{} (0.3, 0.4, 0.3) &{} (0.2, 0.3, 0.3)\\ (0.5, 0.3, 0.7) &{} (0.2, 0.3, 0.5) &{} (0.3, 0.4, 0.3) &{} (0.0, 0.0, 0.0) &{} (0.2, 0.3, 0.7)\\ (0.2, 0.5, 0.7) &{} (0.7, 0.3, 0.5) &{} (0.2, 0.3, 0.3) &{} (0.2, 0.3, 0.7) &{} (0.0, 0.0, 0.0)\\ \end{array} \right] \end{aligned}$$

### Proposition 2

Let ${\mathbb {G}} = (\zeta , \sigma )$ be an *m*-PF graph and $\widetilde{{\mathbb {G}}} = (\widetilde{\zeta }, \widetilde{\sigma })$ be an *m*-PF subgraph of ${\mathbb {G}}$. Then for each $1 \le q \le m$, $P_q \circ CI(\widetilde{{\mathbb {G}}}) \le P_q \circ CI({\mathbb {G}})$, that is, $CI(\widetilde{{\mathbb {G}}})$ will be less than or equal to $CI({\mathbb {G}})$.

We now discuss several particular cases of Proposition 1.

**1** Let ${\mathbb {G}} = (\zeta , \sigma )$ be an *m*-PF graph, and let $\widetilde{{\mathbb {G}}} = (\widetilde{\zeta }, \widetilde{\sigma })$ be a partial *m*-PF subgraph of ${\mathbb {G}}$ that stems from the elimination of a vertex (say *w*) from ${\mathbb {G}}$, i.e., $\widetilde{{\mathbb {G}}} = {\mathbb {G}} \setminus \{w\}$. Then for each $1 \le q \le m$, $P_q \circ CI(\widetilde{{\mathbb {G}}}) < P_q \circ CI({\mathbb {G}})$, that is, $CI(\widetilde{{\mathbb {G}}})$ will be strictly less than $CI({\mathbb {G}})$. Consider the following example.

### Example 3

**Fig. 3:**
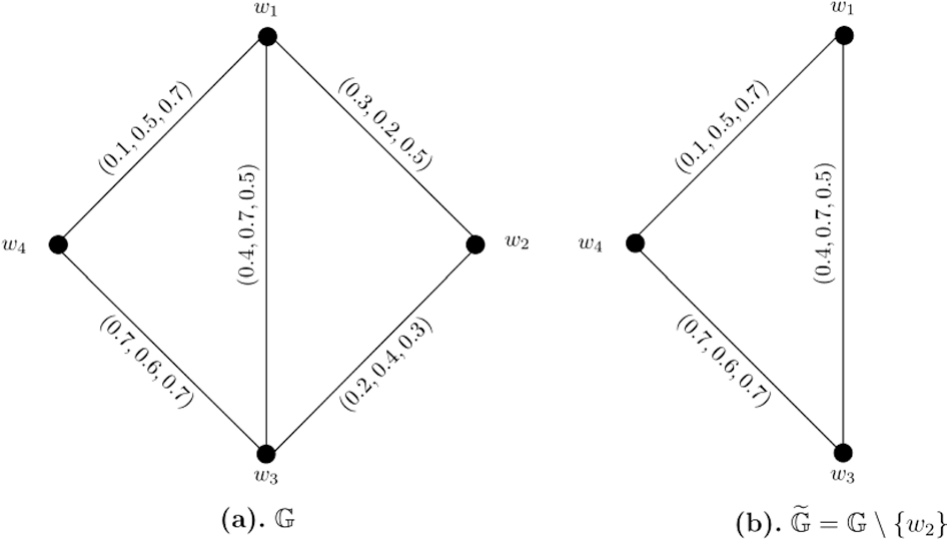
3-PF graph ${\mathbb {G}}$ and partial 3-PF subgraph $\widetilde{{\mathbb {G}}}$ of ${\mathbb {G}}$

Let $W = \{w_1, w_2, w_3, w_4\}$ be a vertex set and $E = \{w_1w_2, w_1w_3, w_1w_4, w_2w_3, w_3w_4\}$ be the set of edges. The corresponding 3-PF graph ${\mathbb {G}} = (\zeta , \sigma )$ is given in Fig. 3a with $\zeta (w_i) = (1, 1, 1)$ for all $i = 1, 2, \ldots , 4$. The strength of connectedness between all pairs of vertices of ${\mathbb {G}}$ are calculated in the following connectivity matrix (3). After calculations, we have $CI({\mathbb {G}}) = (2.1, 3.1, 3.6)$. Now, consider a partial 3-PF subgraph $\widetilde{{\mathbb {G}}} = (\widetilde{\zeta }, \widetilde{\sigma })$ (given in Fig. 3b) of ${\mathbb {G}}$ such that it is obtained after deleting vertex $w_2$ from ${\mathbb {G}}$, i.e., $\widetilde{{\mathbb {G}}} = {\mathbb {G}} \setminus \{w_2\}$. The strength of connectedness between all pairs of vertices of $\widetilde{{\mathbb {G}}}$ are calculated in the connectivity matrix (4). Some computations produce $CI(\widetilde{{\mathbb {G}}}) = (1.5, 1.9, 2.1)$. Clearly, for each $1 \le q \le 3$, $P_q \circ CI(\widetilde{{\mathbb {G}}}) < P_q \circ CI({\mathbb {G}})$, that is to say, $CI(\widetilde{{\mathbb {G}}})$ is strictly smaller than $CI({\mathbb {G}})$.

3 $$\begin{aligned}&\left[ \begin{array}{cccc} (0.0, 0.0, 0.0) &{} (0.3, 0.4, 0.5) &{} (0.4, 0.7, 0.7) &{} (0.4, 0.6, 0.7)\\ (0.3, 0.4, 0.5) &{} (0.0, 0.0, 0.0) &{} (0.3 0.4, 0.5) &{} (0.3, 0.4, 0.5)\\ (0.4, 0.7, 0.7) &{} (0.3 0.4, 0.5) &{} (0.0, 0.0, 0.0) &{} (0.7, 0.6, 0.7)\\ (0.4, 0.6, 0.7) &{} (0.3, 0.4, 0.5) &{} (0.7, 0.6, 0.7) &{} (0.0, 0.0, 0.0)\\ \end{array} \right] \end{aligned}$$

4 $$\begin{aligned}&\left[ \begin{array}{ccc} (0.0, 0.0, 0.0) &{} (0.4, 0.7, 0.7) &{} (0.4, 0.6, 0.7)\\ (0.4, 0.7, 0.7) &{} (0.0, 0.0, 0.0) &{} (0.7 0.6, 0.7)\\ (0.4, 0.6, 0.7) &{} (0.7 0.6, 0.7) &{} (0.0, 0.0, 0.0)\\ \end{array} \right] \end{aligned}$$

**2** Let ${\mathbb {G}} = (\zeta , \sigma )$ be an *m*-PF graph whose crisp underlying graph ${\mathbb {G}}^{\star } = (W, E)$ is a cycle with *wz* as a weakest *m*-PF of ${\mathbb {G}}$ and $\widetilde{{\mathbb {G}}} = (\widetilde{\zeta }, \widetilde{\sigma })$ be a partial *m*-PF subgraph of ${\mathbb {G}}$ such that it is obtained by deleting edge *wz* from ${\mathbb {G}}$, i.e., $\widetilde{{\mathbb {G}}} = {\mathbb {G}} \setminus \{wz\}$. Then for each $1 \le q \le m$, $P_q \circ CI(\widetilde{{\mathbb {G}}}) = P_q \circ CI({\mathbb {G}})$, that is, $CI(\widetilde{{\mathbb {G}}})$ will be equal to $CI({\mathbb {G}})$. Consider the following example.

### Example 4

**Fig. 4:**
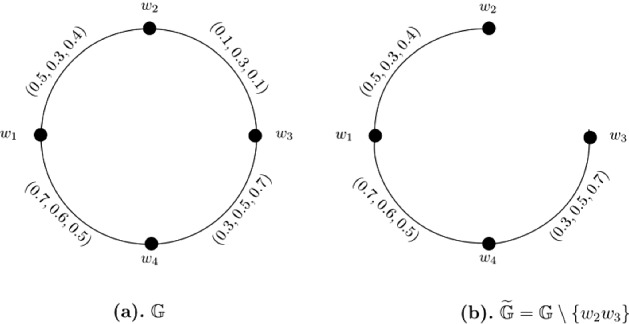
3-PF graph ${\mathbb {G}}$ and partial 3-PF subgraph $\widetilde{{\mathbb {G}}}$ of ${\mathbb {G}}$

Let $W = \{w_1, w_2, w_3, w_4\}$ be a vertex set and $E = \{w_1w_2, w_2w_3, w_3w_4, w_4w_1\}$ be the set of edges. The corresponding 3-PF graph ${\mathbb {G}} = (\zeta , \sigma )$ is given in Fig. 4a with $\zeta (w_i) = (1, 1, 1)$ for all $i = 1, 2, \ldots , 4$. The strength of connectedness between all pairs of vertices of ${\mathbb {G}}$ are calculated in the following connectivity matrix (5). After calculations, we have $CI({\mathbb {G}}) = (2.6, 2.5, 2.9)$. Now, consider a partial 3-PF subgraph $\widetilde{{\mathbb {G}}} = (\widetilde{\zeta }, \widetilde{\sigma })$ (given in Fig. 4b) of ${\mathbb {G}}$ such that it is obtained after deleting edge $w_2w_3$ from ${\mathbb {G}}$, i.e., $\widetilde{{\mathbb {G}}} = {\mathbb {G}} \setminus \{w_2w_3\}$ ($w_2w_3$ is the weakest 3-PF of ${\mathbb {G}}$). The strength of connectedness between all pairs of vertices of $\widetilde{{\mathbb {G}}}$ are calculated in the following connectivity matrix (6). After calculations, we have $CI(\widetilde{{\mathbb {G}}}) = (2.6, 2.5, 2.9)$. Clearly, for each $1 \le q \le 3$, $P_q \circ CI(\widetilde{{\mathbb {G}}}) = P_q \circ CI({\mathbb {G}})$, that is, $CI(\widetilde{{\mathbb {G}}})$ is equal to $CI({\mathbb {G}})$.

5 $$\begin{aligned}&\left[ \begin{array}{cccc} (0.0, 0.0, 0.0) &{} (0.5, 0.3, 0.4) &{} (0.3, 0.5, 0.5) &{} (0.7, 0.6, 0.5)\\ (0.5, 0.3, 0.4) &{} (0.0, 0.0, 0.0) &{} (0.3 0.3, 0.4) &{} (0.5, 0.3, 0.4)\\ (0.3, 0.5, 0.5) &{} (0.3 0.3, 0.4) &{} (0.0, 0.0, 0.0) &{} (0.3, 0.5, 0.7)\\ (0.7, 0.6, 0.5) &{} (0.5, 0.3, 0.4) &{} (0.3, 0.5, 0.7) &{} (0.0, 0.0, 0.0)\\ \end{array} \right] \end{aligned}$$

6 $$\begin{aligned}&\left[ \begin{array}{cccc} (0.0, 0.0, 0.0) &{} (0.5, 0.3, 0.4) &{} (0.3, 0.5, 0.5) &{} (0.7, 0.6, 0.5)\\ (0.5, 0.3, 0.4) &{} (0.0, 0.0, 0.0) &{} (0.3 0.3, 0.4) &{} (0.5, 0.3, 0.4)\\ (0.3, 0.5, 0.5) &{} (0.3 0.3, 0.4) &{} (0.0, 0.0, 0.0) &{} (0.3, 0.5, 0.7)\\ (0.7, 0.6, 0.5) &{} (0.5, 0.3, 0.4) &{} (0.3, 0.5, 0.7) &{} (0.0, 0.0, 0.0)\\ \end{array} \right] \end{aligned}$$

### Theorem 1

Let ${\mathbb {G}}_1 = (\zeta _1, \sigma _1)$ and ${\mathbb {G}}_2 = (\zeta _2, \sigma _2)$ be two homomorphic *m*-PF graphs. Then for each $1 \le q \le m$, $P_q \circ CI({\mathbb {G}}_1) \le P_q \circ CI({\mathbb {G}}_2)$, that is, $CI({\mathbb {G}}_1)$ will be less than or equal to $CI({\mathbb {G}}_2)$.

### Proof

Let ${\mathbb {G}}_1 = (\zeta _1, \sigma _1)$ and ${\mathbb {G}}_2 = (\zeta _2, \sigma _2)$ be two homomorphic *m*-PF graphs. Then there exists a mapping $\xi : W_1 \longrightarrow W_2$ such that for each $1 \le q \le m$, $P_q \circ \zeta _1(w) \le P_q \circ \zeta _2(\xi (w))$ for all $w \in W_1$ and $P_q \circ \sigma _1(wz) \le P_q \circ \sigma _2(\xi (w)\xi (z))$ for all $wz \in E_1$. Also, the homomorphism between ${\mathbb {G}}_1$ and ${\mathbb {G}}_2$ implies that the strength of any strongest path between any two vertices *w* and *z* in ${\mathbb {G}}_1$ is less than or equal to that between $\xi (w)$ and $\xi (z)$ in ${\mathbb {G}}_2$. Therefore, we have for each $1 \le q \le m$, $P_q \circ CONN_{{\mathbb {G}}_1}(w, z) \le P_q \circ CONN_{{\mathbb {G}}_2}(\xi (w), \xi (z))$ for all $w, z \in W_1$. This implies that for each $1 \le q \le m$, $\sum _{w, z \in W_1}P_q \circ \zeta _1(w)P_q \circ \zeta _1(z)P_q \circ CONN_{{\mathbb {G}}_1}(w, z) \le \sum _{\xi (w), \xi (z) \in W_2}P_q \circ \zeta _2(\xi (w))P_q \circ \zeta _2(\xi (z))P_q \circ CONN_{{\mathbb {G}}_2}(\xi (w), \xi (z))$. This implies that for each $1 \le q \le m$, $P_q \circ CI({\mathbb {G}}_1) \le P_q \circ CI({\mathbb {G}}_2)$. Thus, $CI({\mathbb {G}}_1)$ is less than or equal to $CI({\mathbb {G}}_1)$. $\square$

If ${\mathbb {G}}_1$ and ${\mathbb {G}}_2$ are isomorphic to each other, the equality between $CI({\mathbb {G}}_1)$ and $CI({\mathbb {G}}_2)$ holds. The connectivity indices of two isomorphic *m*-PF graphs are described by the following theorem.

### Theorem 2

Let ${\mathbb {G}}_1 = (\zeta _1, \sigma _1)$ and ${\mathbb {G}}_2 = (\zeta _2, \sigma _2)$ be two isomorphic *m*-PF graphs. Then for each $1 \le q \le m$, $P_q \circ CI({\mathbb {G}}_1) = P_q \circ CI({\mathbb {G}}_2)$, that is, $CI({\mathbb {G}}_1)$ will be equal to $CI({\mathbb {G}}_2)$.

### Proof

Let ${\mathbb {G}}_1 = (\zeta _1, \sigma _1)$ and ${\mathbb {G}}_2 = (\zeta _2, \sigma _2)$ be two isomorphic *m*-PF graphs. Then there exists a bijective mapping $\xi : W_1 \longrightarrow W_2$ such that for each $1 \le q \le m$, $P_q \circ \zeta _1(w) = P_q \circ \zeta _2(\xi (w))$ for all $w \in W_1$ and $P_q \circ \sigma _1(wz) = P_q \circ \sigma _2(\xi (w)\xi (z))$ for all $wz \in E_1$. Also, the isomorphism between ${\mathbb {G}}_1$ and ${\mathbb {G}}_2$ implies that the strength of any strongest path between any two vertices *w* and *z* in ${\mathbb {G}}_1$ is equal to that between $\xi (w)$ and $\xi (z)$ in ${\mathbb {G}}_2$. Therefore, we have for each $1 \le q \le m$, $P_q \circ CONN_{{\mathbb {G}}_1}(w, z) = P_q \circ CONN_{{\mathbb {G}}_2}(\xi (w), \xi (z))$ for all $w, z \in W_1$. This implies that for each $1 \le q \le m$, $\sum _{w, z \in W_1}P_q \circ \zeta _1(w)P_q \circ \zeta _1(z)P_q \circ CONN_{{\mathbb {G}}_1}(w, z) = \sum _{\xi (w), \xi (z) \in W_2}P_q \circ \zeta _2(\xi (w))P_q \circ \zeta _2(\xi (z))P_q \circ CONN_{{\mathbb {G}}_2}(\xi (w), \xi (z))$. This implies that for each $1 \le q \le m$, $P_q \circ CI({\mathbb {G}}_1) = P_q \circ CI({\mathbb {G}}_2)$. Thus, $CI({\mathbb {G}}_1)$ equals $CI({\mathbb {G}}_1)$. $\square$

## Bounds for the connectivity index of *m*-PF graphs

We now describe several bounds for the connectivity index of *m*-PF graphs in this section. Also, we examine the connectivity index of a complete *m*-PF graph by various theorems. The complete *m*-PF graph will have the maximum connectivity among all *m*-PF graphs on a fixed support (having the same vertex set), as shown in the next result.

### Theorem 3

Let ${\mathbb {G}} = (\zeta , \sigma )$ be an *m*-PF graph with $|W| = n$. If $\acute{{\mathbb {G}}} = (\acute{\zeta }, \acute{\sigma })$ is the completion of ${\mathbb {G}}$ spanned by its vertex set, then for each $1 \le q \le m$, $0 \le P_q \circ CI({\mathbb {G}}) \le P_q \circ CI(\acute{{\mathbb {G}}})$.

### Proof

Let ${\mathbb {G}} = (\zeta , \sigma )$ be an *m*-PF graph with $|W| = n$ and $\acute{{\mathbb {G}}} = (\acute{\zeta }, \acute{\sigma })$ be the completion of ${\mathbb {G}}$ spanned by its vertex set, i.e., $|W| = |\acute{W}|$ and $P_q \circ \zeta (w) = P_q \circ \acute{\zeta }(w)$ for each $1 \le q \le m$. We have three cases as follows:

**Case 1** If ${\mathbb {G}}$ is a trivial *m*-PF graph ($|E| = 0$) with $|W| = n$ and $n = 1$. Then its completion $\acute{{\mathbb {G}}}$ is also a trivial *m*-PF graph. Obviously, for each $1 \le q \le m$, $P_q \circ CI({\mathbb {G}}) = 0 = P_q \circ CI(\acute{{\mathbb {G}}})$.

**Case 2** If ${\mathbb {G}}$ is a trivial *m*-PF graph ($|E| = 0$) with $|W| = n$ and $n > 1$. Then for each $1 \le q \le m$, $P_q \circ CONN_{{\mathbb {G}}}(w, z) = 0$ for all pairs of vertices of ${\mathbb {G}}$. This means that for each $1 \le q \le m$, $P_q \circ CI({\mathbb {G}}) = 0$. Since, $\acute{{\mathbb {G}}}$ is the completion of ${\mathbb {G}}$ spanned by its vertex set, therefore, for each $1 \le q \le m$, $P_q \circ CONN_{\acute{{\mathbb {G}}}}(w, z) \ge 0$ for all pairs of vertices of $\acute{{\mathbb {G}}}$. This shows that for each $1 \le q \le m$, $P_q \circ CI(\acute{{\mathbb {G}}}) \ge 0$. Thus, for each $1 \le q \le m$, $P_q \circ CI({\mathbb {G}}) \le P_q \circ CI(\acute{{\mathbb {G}}})$.

**Case 3** If ${\mathbb {G}}$ is a non-trivial *m*-PF graph ($|E| > 0$) with $|W| = n$. Then for each $1 \le q \le m$, $P_q \circ \sigma (wz) \ge 0$ for all $w, z \in W$. This implies that for each $1 \le q \le m$, $P_q \circ CONN_{{\mathbb {G}}}(w, z) \ge 0$. Since, $\acute{{\mathbb {G}}} = (\acute{\zeta }, \acute{\sigma })$ is the completion of ${\mathbb {G}}$ spanned by its vertex set, therefore, for each $1 \le q \le m$, $P_q \circ CONN_{{\mathbb {G}}}(w, z) \le P_q \circ CONN_{\acute{{\mathbb {G}}}}(w, z)$ for all $w, z \in W$. This implies that for each $1 \le q \le m$, $\sum _{w, z \in W}P_q \circ \zeta (w)P_q \circ \zeta (z)P_q \circ CONN_{{\mathbb {G}}}(w, z) \le \sum _{w, z \in \acute{W}}P_q \circ \acute{\zeta }(w)P_q \circ \acute{\zeta }(z)P_q \circ CONN_{\acute{{\mathbb {G}}}}(w, z)$ for all $w, z \in \acute{W}$. Hence, for each $1 \le q \le m$, $P_q \circ CI({\mathbb {G}}) \le P_q \circ CI(\acute{{\mathbb {G}}})$.

Thus, in all cases for each $1 \le q \le m$, $0 \le P_q \circ CI({\mathbb {G}}) \le P_q \circ CI(\acute{{\mathbb {G}}})$ holds true. $\square$

### Theorem 4

Let ${\mathbb {G}} = (\zeta , \sigma )$ be a complete *m*-PF graph with $|W| = n$ such that $P_1 \circ \zeta (w_i) \le P_2 \circ \zeta (w_i) \le \ldots \le P_m \circ \zeta (w_i)$ for $i = 1, 2, \ldots , n$ and $P_q \circ \zeta (w_1) \le P_q \circ \zeta (w_2) \le \ldots \le P_q \circ \zeta (w_n)$ for each $1 \le q \le m$. Then $P_q \circ CI({\mathbb {G}}) = \sum _{i = 1}^{n - 1}(P_q \circ \zeta (w_i))^{2}\sum _{k = i + 1}^{n}P_q \circ \zeta (w_k)$ for each $1 \le q \le m$.

### Proof

Let ${\mathbb {G}} = (\zeta , \sigma )$ be a complete *m*-PF graph with $|W| = n$ such that $P_1 \circ \zeta (w_i) \le P_2 \circ \zeta (w_i) \le \ldots \le P_m \circ \zeta (w_i)$ for $i = 1, 2, \ldots , n$ and $P_q \circ \zeta (w_1) \le P_q \circ \zeta (w_2) \le \ldots \le P_q \circ \zeta (w_n)$ for each $1 \le q \le m$. It is obvious that $w_1$ is the vertex of ${\mathbb {G}}$ such that $P_q \circ \zeta (w_1) \le P_q \circ \zeta (w_i)$ for $i = 2, 3, \ldots , n$ and for each $1 \le q \le m$, i.e., $w_1$ is of least membership value. Also, in complete *m*-PF graph $P_q \circ CONN_{{\mathbb {G}}}(w, z) = P_q \circ \sigma (wz) = \inf \{P_q \circ \zeta (w), P_q \circ \zeta (z)\}$ for all $w, z \in W$ and for each $1 \le q \le m$. This implies that $P_q \circ \sigma (w_1w_i) = P_q \circ \zeta (w_1)$ and $P_q \circ CONN_{{\mathbb {G}}}(w_1, w_i) = P_q \circ \zeta (w_1)$ for $i = 2, 3, \ldots , n$ and for each $1 \le q \le m$. Now, consider $P_q \circ CI({\mathbb {G}}) = \sum _{w_i, w_k \in W w_i \ne w_k} P_q \circ \zeta (w_i) P_q \circ \zeta (w_k) P_q \circ CONN_{{\mathbb {G}}}(w_i, w_k)$ for each $1 \le q \le m$. Consider $P_q \circ \zeta (w_i)P_q \circ \zeta (w_k)P_q \circ CONN_{{\mathbb {G}}}(w_i, w_k)$ for $i = 1$ and $k = 2, 3, \ldots , n$. Since, $w_1$ is of least membership value and ${\mathbb {G}}$ is complete *m*-PF graph. Therefore, $P_q \circ \zeta (w_1)P_q \circ \zeta (w_k)P_q \circ CONN_{{\mathbb {G}}}(w_1, w_k) = P_q \circ \zeta (w_1)P_q \circ \zeta (w_k)P_q \circ \zeta (w_1)$ for each $1 \le q \le m$. Taking summation over *k*, we have

7 $$\begin{aligned}&\sum _{k = 2}^{n} P_q \circ \zeta (w_1)P_q \circ \zeta (w_k)P_q \circ CONN_{{\mathbb {G}}}(w_1, w_k) \nonumber \\&\quad = (P_q \circ \zeta (w_1))^2 \sum _{k = 2}^{n}P_q \circ \zeta (w_k), \end{aligned}$$

for each $1 \le q \le m$. Consider $P_q \circ \zeta (w_i)P_q \circ \zeta (w_k)P_q \circ CONN_{{\mathbb {G}}}(w_i, w_k)$ for $i = 2, 3, \ldots , n$ and $k = i + 1$ ($k = 3, 4, \ldots , n$). Since, $i < k$ and ${\mathbb {G}}$ is complete *m*-PF graph. Therefore, $P_q \circ \zeta (w_i)P_q \circ \zeta (w_k)P_q \circ CONN_{{\mathbb {G}}}(w_i, w_k) = P_q \circ \zeta (w_i)P_q \circ \zeta (w_k)P_q \circ \zeta (w_i)$ for each $1 \le q \le m$. Taking summation over *i* and *k*, we have

8 $$\begin{aligned}&\sum _{i = 2}^{n}\sum _{k = i + 1}^{n} P_q \circ \zeta (w_i)P_q \circ \zeta (w_k)P_q \circ CONN_{{\mathbb {G}}}(w_i, w_k) \nonumber \\&\quad = \sum _{i = 2}^{n}(P_q \circ \zeta (w_i))^2 \sum _{k = i + 1}^{n}P_q \circ \zeta (w_k), \end{aligned}$$

for each $1 \le q \le m$. Adding Eqs. 7 and 8, we have

$$\begin{aligned}&\sum _{w_i, w_k \in W} P_q \circ \zeta (w_1)P_q \circ \zeta (w_k)P_q \circ CONN_{{\mathbb {G}}}(w_1, w_k) \\&\quad = (P_q \circ \zeta (w_1))^2 \sum _{k = 2}^{n}P_q \circ \zeta (w_k)\\&\qquad + \sum _{i = 2}^{n}(P_q \circ \zeta (w_i))^2 \sum _{k = i + 1}^{n}P_q \circ \zeta (w_k), \end{aligned}$$

for each $1 \le q \le m$. By combining both terms on R.H.S. of above equation, we have

$$\begin{aligned} P_q \circ CI({\mathbb {G}})= & {} \sum _{i = 1}^{1}(P_q \circ \zeta (w_i))^2 \sum _{k = i + 1}^{n}P_q \circ \zeta (w_k), \end{aligned}$$

for each $1 \le q \le m$. This completes the proof. $\square$

### Example 5

**Fig. 5:**
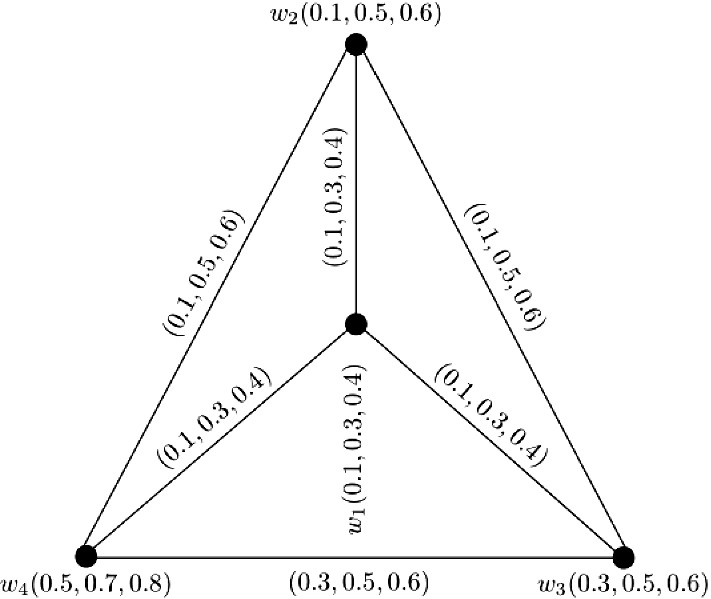
Complete 3-PF graph ${\mathbb {G}}$

Let $W = \{w_1, w_2, w_3, w_4\}$ be a vertex set and $E = \{w_1w_2, w_1w_3, w_1w_4, w_2w_3, w_2w_4, w_3w_4\}$ be the set of edges. The corresponding 3-PF graph ${\mathbb {G}} = (\zeta , \sigma )$ is given in Fig. 5 with $\zeta (w_1) = (0.1, 0.3, 0.4)$, $\zeta (w_2) = (0.1, 0.5, 0.6)$, $\zeta (w_3) = (0.3, 0.5, 0.6)$, and $\zeta (w_4) = (0.5, 0.7, 0.8)$. Clearly, ${\mathbb {G}}$ is a complete 3-PF graph satisfying $P_1 \circ \zeta (w_i) \le P_2 \circ \zeta (w_i) \le P_3 \circ \zeta (w_i)$ for $i = 1, 2, 3, 4$ and $P_q \circ \zeta (w_1) \le P_q \circ \zeta (w_2) \le P_q \circ \zeta (w_3) \le P_q \circ \zeta (w_4)$ for each $1 \le q \le 3$.

Therefore, we can calculate $CI({\mathbb {G}})$ by using Theorem 4 as

$$\begin{aligned} CI({\mathbb {G}})= & {} (P_1 \circ CI({\mathbb {G}}), P_2 \circ CI({\mathbb {G}}), P_3 \circ CI({\mathbb {G}}))\\= & {} \left( \sum _{i = 1}^{n - 1}(P_1 \circ \zeta (w_i))^{2}\sum _{k = i + 1}^{n}P_1 \circ \zeta (w_k), \sum _{i = 1}^{n - 1}(P_2 \circ \zeta (w_i))^{2}\sum _{k = i + 1}^{n}P_2 \circ \zeta (w_k),\right. \\&\left. \sum _{i = 1}^{n - 1}(P_3 \circ \zeta (w_i))^{2}\sum _{k = i + 1}^{n}P_3 \circ \zeta (w_k)\right) . \end{aligned}$$

Here,

$$\begin{aligned} P_1 \circ CI({\mathbb {G}})= & {} \sum _{i = 1}^{3}(P_1 \circ \zeta (w_i))^{2}\sum _{k = i + 1}^{4}P_1 \circ \zeta (w_k)\\= & {} (P_1 \circ \zeta (w_1))^{2}P_1 \circ \zeta (w_2) + (P_1 \circ \zeta (w_1))^{2}P_1 \circ \zeta (w_3)\\&+ (P_1 \circ \zeta (w_1))^{2}P_1 \circ \zeta (w_4) + (P_1 \circ \zeta (w_2))^{2}P_1 \circ \zeta (w_3)\\&+ (P_1 \circ \zeta (w_2))^{2}P_1 \circ \zeta (w_4) + (P_1 \circ \zeta (w_3))^{2}P_1 \circ \zeta (w_4)\\= & {} (0.1)^{2}0.1 + (0.1)^{2}0.3 + (0.1)^{2}0.5\\&+ (0.1)^{2}0.3 + (0.1)^{2}0.5 + (0.3)^{2}0.5\\= & {} 0.062. \end{aligned}$$

Similarly, we obtain $P_2 \circ CI({\mathbb {G}}) = 0.628$ and $P_3 \circ CI({\mathbb {G}}) = 1.112$. After calculations, we have

$$\begin{aligned} CI({\mathbb {G}})= & {} (0.062, 0.628, 1.112). \end{aligned}$$

Note that $P_q \circ \zeta (w_1) \le P_q \circ \zeta (w_2) \le \ldots \le P_q \circ \zeta (w_n)$ for each $1 \le q \le m$ implies $P_1 \circ \zeta (w_i) \le P_2 \circ \zeta (w_i) \le \ldots \le P_m \circ \zeta (w_i)$ for $i = 1, 2, \ldots , n$. Therefore, if a complete *m*-PF graph ${\mathbb {G}} = (\zeta , \sigma )$ does not satisfy the condition $P_q \circ \zeta (w_1) \le P_q \circ \zeta (w_2) \le \ldots \le P_q \circ \zeta (w_n)$ for each $1 \le q \le m$, then Theorem 4 need not be true.

### Theorem 5

Let ${\mathbb {G}} = (\zeta , \sigma )$ be a complete bipartite *m*-PF graph with $|W| = |W_1 \cup W_2|= n$. Also, let $w_1 = \{w_1, w_2, \ldots , w_m\}$ and $w_2 = \{w_{m + 1}, w_{m + 2}, \ldots , w_n\}$ such that $P_1 \circ \zeta (w_i) \le P_2 \circ \zeta (w_i) \le \ldots \le P_m \circ \zeta (w_i)$ for $i = 1, 2, \ldots , n$ and $P_q \circ \zeta (w_1) \le P_q \circ \zeta (w_2) \le \ldots \le P_q \circ \zeta (w_n)$ for each $1 \le q \le m$. Then $P_q \circ CI({\mathbb {G}}) = \sum _{i = 1}^{m}(P_q \circ \zeta (w_i))^{2}\sum _{k = i + 1}^{n}P_q \circ \zeta (w_k) + P_q \circ \zeta (w_m) \sum _{i = m + 1}^{n - 1}P_q \circ \zeta (w_i)\sum _{k = i + 1}^{n}P_q \circ \zeta (w_k)$ for each $1 \le q \le m$.

### Proof

Let ${\mathbb {G}} = (\zeta , \sigma )$ be a complete bipartite *m*-PF graph with $|W| = |W_1 \cup W_2|= n$. Also, let $w_1 = \{w_1, w_2, \ldots , w_m\}$ and $w_2 = \{w_{m + 1}, w_{m + 2}, \ldots , w_n\}$ such that $P_1 \circ \zeta (w_i) \le P_2 \circ \zeta (w_i) \le \ldots \le P_m \circ \zeta (w_i)$ for $i = 1, 2, \ldots , n$ and $P_q \circ \zeta (w_1) \le P_q \circ \zeta (w_2) \le \ldots \le P_q \circ \zeta (w_n)$ for each $1 \le q \le m$. It is obvious that $w_1$ is the vertex of ${\mathbb {G}}$ such that $P_q \circ \zeta (w_1) \le P_q \circ \zeta (w_i)$ for $i = 2, 3, \ldots , n$ and for each $1 \le q \le m$, i.e., $w_1$ is of least membership value. Since, ${\mathbb {G}}$ is a complete *m*-PF graph. This implies that $P_q \circ \sigma (wz) = 0$ for each $1 \le q \le m$, if $w, z \in W_1$ or $w, z \in W_2$, and $P_q \circ \sigma (wz) = \inf \{P_q \circ \zeta (w), P_q \circ \zeta (z)\}$ for each $1 \le q \le m$, if $w \in W_1$ and $z \in W_2$ or $w \in W_2$ and $z \in W_1$. Now, consider $P_q \circ CI({\mathbb {G}}) = \sum _{w_i, w_k \in W} P_q \circ \zeta (w_i) P_q \circ \zeta (w_k) P_q \circ CONN_{{\mathbb {G}}}(w_i, w_k)$ for each $1 \le q \le m$. Consider $P_q \circ \zeta (w_i)P_q \circ \zeta (w_k)P_q \circ CONN_{{\mathbb {G}}}(w_i, w_k)$ for $i = 1$ and $k = 2, 3, \ldots , n$. Since, $w_1$ is of least membership value and ${\mathbb {G}}$ is complete bipartite *m*-PF graph. Therefore, $P_q \circ \zeta (w_1)P_q \circ \zeta (w_k)P_q \circ CONN_{{\mathbb {G}}}(w_1, w_k) = P_q \circ \zeta (w_1)P_q \circ \zeta (w_k)P_q \circ \zeta (w_1)$ for each $1 \le q \le m$. Taking summation over *k*, we have

9 $$\begin{aligned}&\sum _{k = 2}^{n} P_q \circ \zeta (w_1)P_q \circ \zeta (w_k)P_q \circ CONN_{{\mathbb {G}}}(w_1, w_k) = (P_q \circ \zeta (w_1))^2 \sum _{k = 2}^{n}P_q \circ \zeta (w_k), \end{aligned}$$

for each $1 \le q \le m$. Consider $P_q \circ \zeta (w_i)P_q \circ \zeta (w_k)P_q \circ CONN_{{\mathbb {G}}}(w_i, w_k)$ for $i = 2, 3, \ldots , m$ and $k = i + 1$ ($k = 3, 4, \ldots , n$). Since, $i < k$ and ${\mathbb {G}}$ is complete bipartite *m*-PFG. Therefore, $P_q \circ \zeta (w_i)P_q \circ \zeta (w_k)P_q \circ CONN_{{\mathbb {G}}}(w_i, w_k) = P_q \circ \zeta (w_i)P_q \circ \zeta (w_k)P_q \circ \zeta (w_i)$ for each $1 \le q \le m$. Taking summation over *i* and *k*, we have

10 $$\begin{aligned}&\sum _{i = 2}^{m}\sum _{k = i + 1}^{n} P_q \circ \zeta (w_i)P_q \circ \zeta (w_k)P_q \circ CONN_{{\mathbb {G}}}(w_i, w_k) = \sum _{i = 2}^{m}(P_q \circ \zeta (w_i))^2 \sum _{k = i + 1}^{n}P_q \circ \zeta (w_k), \end{aligned}$$

for each $1 \le q \le m$. Consider $P_q \circ \zeta (w_i)P_q \circ \zeta (w_k)P_q \circ CONN_{{\mathbb {G}}}(w_i, w_k)$ for $m< i < n$ ($i = m + 1, m + 2, \ldots , n - 1$) and $k = i + 1$ ($k = m + 2, m + 3, \ldots , n$). Since, $m < k$ and ${\mathbb {G}}$ is complete bipartite *m*-PF graph. Therefore, $P_q \circ \zeta (w_i)P_q \circ \zeta (w_k)P_q \circ CONN_{{\mathbb {G}}}(w_i, w_k) = P_q \circ \zeta (w_i)P_q \circ \zeta (w_k)P_q \circ \zeta (w_m)$. Taking summation over *i* and *k*, we have

11 $$\begin{aligned}&\sum _{i = m + 1}^{n - 1}\sum _{k = i + 1}^{n} P_q \circ \zeta (w_i)P_q \circ \zeta (w_k)P_q \circ CONN_{{\mathbb {G}}}(w_i, w_k) \nonumber \\&\quad = P_q \circ \zeta (w_m) \sum _{i = m + 1}^{n - 1}P_q \circ \zeta (w_i) \sum _{k = i + 1}^{n}P_q \circ \zeta (w_k), \end{aligned}$$

for each $1 \le q \le m$. Adding Eqs. 9, 10 and 11, we have

$$\begin{aligned}&\sum _{w_i, w_k \in W} P_q \circ \zeta (w_1)P_q \circ \zeta (w_k)P_q \circ CONN_{{\mathbb {G}}}(w_1, w_k) \\&\quad = (P_q \circ \zeta (w_1))^2 \sum _{k = 2}^{n}P_q \circ \zeta (w_k)\\&\qquad + \sum _{i = 2}^{m}(P_q \circ \zeta (w_i))^2 \sum _{k = i + 1}^{n}P_q \circ \zeta (w_k)\\&\qquad + P_q \circ \zeta (w_m) \sum _{i = m + 1}^{n - 1}P_q \circ 
\zeta (w_i) \sum _{k = i + 1}^{n}P_q \circ \zeta (w_k), \end{aligned}$$

for each $1 \le q \le m$. By combining first two terms on R.H.S. of above equation, we have

$$\begin{aligned} P_q \circ CI({\mathbb {G}})= & {} \sum _{i = 1}^{m}(P_q \circ \zeta (w_i))^2 \sum _{k = i + 1}^{n}P_q \circ \zeta (w_k) + P_q \circ \zeta (w_m) \sum _{i = m + 1}^{n - 1}P_q \circ \zeta (w_i)\\&\sum _{k = i + 1}^{n}P_q \circ \zeta (w_k), \end{aligned}$$

for each $1 \le q \le m$. This completes the proof. $\square$

### Example 6

**Fig. 6:**
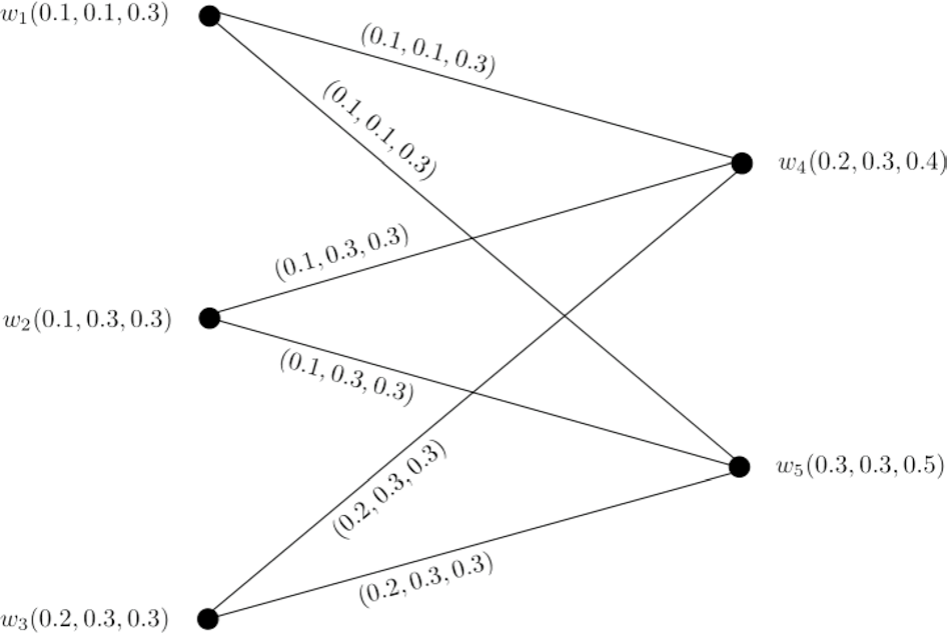
Complete bipartite 3-PF graph ${\mathbb {G}}$

Let $W = \{w_1, w_2, w_3, w_4, w_5\}$ be a vertex set and $E = \{w_1w_4, w_1w_5, w_2w_4, w_2w_5, w_3w_4, w_3w_5\}$ be the set of edges. The corresponding 3-PF graph ${\mathbb {G}} = (\zeta , \sigma )$ is given in Fig. 6 with $\zeta (w_1) = (0.1, 0.1, 0.3)$, $\zeta (w_2) = (0.1, 0.3, 0.3)$, $\zeta (w_3) = (0.2, 0.3, 0.3)$, $\zeta (w_4) = (0.2, 0.3, 0.4)$, and $w_5 = (0.3, 0.3, 0.5)$. Clearly, ${\mathbb {G}}$ is a complete bipartite 3-PF graph satisfying $P_1 \circ \zeta (w_i) \le P_2 \circ \zeta (w_i) \le P_3 \circ \zeta (w_i)$ for $i = 1, 2, \ldots , 5$ and $P_q \circ \zeta (w_1) \le P_q \circ \zeta (w_2) \le \ldots \le P_q \circ \zeta (w_5)$ for each $1 \le q \le 3$.

Therefore, we can calculate $CI({\mathbb {G}})$ by using Theorem 5 as

$$\begin{aligned} CI({\mathbb {G}})= & {} (P_1 \circ CI({\mathbb {G}}), P_2 \circ CI({\mathbb {G}}), P_3 \circ CI({\mathbb {G}}))\\= & {} \left( \sum _{i = 1}^{m}(P_1 \circ \zeta (w_i))^{2}\sum _{k = i + 1}^{n}P_1 \circ \zeta (w_k) + P_1 \circ \zeta (w_m) \sum _{i = m + 1}^{n - 1}P_1 \circ \zeta (w_i) \right. \\&\sum _{k = i + 1}^{n}P_1 \circ \zeta (w_k),\\&\sum _{i = 1}^{m}(P_2 \circ \zeta (w_i))^{2}\sum _{k = i + 1}^{n}P_2 \circ \zeta (w_k) + P_2 \circ \zeta (w_m) \sum _{i = m + 1}^{n - 1}P_2 \circ \zeta (w_i) \\&\sum _{k = i + 1}^{n}P_2 \circ \zeta (w_k),\\&\sum _{i = 1}^{m}(P_3 \circ \zeta (w_i))^{2}\sum _{k = i + 1}^{n}P_3 \circ \zeta (w_k) + P_3 \circ \zeta (w_m) \sum _{i = m + 1}^{n - 1}P_3 \circ \zeta (w_i) \\&\left. \sum _{k = i + 1}^{n}P_3 \circ \zeta (w_k)\right) . \end{aligned}$$

Here,

$$\begin{aligned} P_1 \circ CI({\mathbb {G}})= & {} \sum _{i = 1}^{3}(P_1 \circ \zeta (w_i))^{2}\sum _{k = i + 1}^{5}P_1 \circ \zeta (w_k) + P_1 \circ \zeta (w_3) \sum _{i = 3 + 1}^{5 - 1}P_1 \circ \zeta (w_i) \\&\sum _{k = i + 1}^{5}P_1 \circ \zeta (w_k)\\= & {} (P_1 \circ \zeta (w_1))^{2}\sum _{k = 2}^{5}P_1 \circ \zeta (w_k) + P_1 \circ \zeta (w_2))^{2}\\&\sum _{k = 3}^{5}P_1 \circ \zeta (w_k)\\&+ P_1 \circ \zeta (w_3))^{2}\sum _{k = 4}^{5}P_1 \circ \zeta (w_k) \\&+ P_1 \circ \zeta (w_3)P_1 \circ \zeta (w_4)P_1 \circ \zeta (w_5)\\= & {} (P_1 \circ \zeta (w_1))^{2}P_1 \circ \zeta (w_2) + (P_1 \circ \zeta (w_1))^{2}P_1 \circ \zeta (w_3) \\&+ (P_1 \circ \zeta (w_1))^{2}P_1 \circ \zeta (w_4)\\&+ (P_1 \circ \zeta (w_1))^{2}P_1 \circ \zeta (w_5) + (P_1 \circ \zeta (w_2))^{2}P_1 \circ \zeta (w_3) \\&+ (P_1 \circ \zeta (w_2))^{2}P_1 \circ \zeta (w_4)\\&+ (P_1 \circ \zeta (w_2))^{2}P_1 \circ \zeta (w_5) + (P_1 \circ \zeta (w_3))^{2}P_1 \circ \zeta (w_4)\\&+ (P_1 \circ \zeta (w_3))^{2}P_1 \circ \zeta (w_5)\\&+ P_1 \circ \zeta (w_3)P_1 \circ \zeta (w_4)P_1 \circ \zeta (w_5)\\= & {} (0.1)^{2}0.1 + (0.1)^{2}0.2 + (0.1)^{2}0.2 + (0.1)^{2}0.3 + (0.1)^{2}0.2\\&+ (0.1)^{2}0.2 + (0.1)^{2}0.3 + (0.2)^{2}0.2 + (0.2)^{2}0.3 + (0.2)(0.2)(0.3)\\= & {} 0.008 + 0.007 + 0.02 + 0.012\\= & {} 0.047. \end{aligned}$$

Similarly, we obtain $P_2 \circ CI({\mathbb {G}}) = 0.174$ and $P_3 \circ CI({\mathbb {G}}) = 0.384$. After calculations, we have

$$\begin{aligned} CI({\mathbb {G}})= & {} (0.047, 0.174, 0.384). \end{aligned}$$

### Theorem 6

Let ${\mathbb {G}} = (\zeta , \sigma )$ be a wheel *m*-PF graph with $|W| = n$ such that $P_1 \circ \zeta (w_i) \le P_2 \circ \zeta (w_i) \le \ldots \le P_m \circ \zeta (w_i)$ for $i = 1, 2, \ldots , n$ and $P_q \circ \zeta (w_1) \le P_q \circ \zeta (w_2) \le \ldots \le P_q \circ \zeta (w_n)$ for each $1 \le q \le m$. Also, let $w_1$ be the center vertex of ${\mathbb {G}}$ and for any edge $wz \in E$, $P_q \circ \sigma (wz) = \inf \{P_q \circ \zeta (w), P_q \circ \zeta (z)\}$ for each $1 \le q \le m$. Then $P_q \circ CI({\mathbb {G}}) = \sum _{i = 1}^{n - 1}(P_q \circ \zeta (w_i))^{2}\sum _{k = i + 1}^{n}P_q \circ \zeta (w_k)$ for each $1 \le q \le m$.

### Proof

Let ${\mathbb {G}} = (\zeta , \sigma )$ be a wheel *m*-PF graph with $|W| = n$ such that $P_1 \circ \zeta (w_i) \le P_2 \circ \zeta (w_i) \le \ldots \le P_m \circ \zeta (w_i)$ for $i = 1, 2, \ldots , n$ and $P_q \circ \zeta (w_1) \le P_q \circ \zeta (w_2) \le \ldots \le P_q \circ \zeta (w_n)$ for each $1 \le q \le m$. Also, let $w_1$ be the center vertex of ${\mathbb {G}}$ and for any edge $wz \in E$, $P_q \circ \sigma (wz) = \inf \{P_q \circ \zeta (w), P_q \circ \zeta (z)\}$ for each $1 \le q \le m$. It is obvious that $w_1$ is the vertex of ${\mathbb {G}}$ such that $P_q \circ \zeta (w_1) \le P_q \circ \zeta (w_i)$ for $i = 2, 3, \ldots , n$ and for each $1 \le q \le m$, i.e., $w_1$ is of least membership value. Now, consider $P_q \circ CI({\mathbb {G}}) = \sum _{w_i, w_k \in W w_i \ne w_k} P_q \circ \zeta (w_i) P_q \circ \zeta (w_k) P_q \circ CONN_{{\mathbb {G}}}(w_i, w_k)$ for each $1 \le q \le m$. Consider $P_q \circ \zeta (w_i)P_q \circ \zeta (w_k)P_q \circ CONN_{{\mathbb {G}}}(w_i, w_k)$ for $i = 1$ and $k = 2, 3, \ldots , n$. Since, $w_1$ is of least membership value and for any edge $wz \in E$, $P_q \circ \sigma (wz) = \inf \{P_q \circ \zeta (w), P_q \circ \zeta (z)\}$ for each $1 \le q \le m$. Therefore, $P_q \circ \zeta (w_1)P_q \circ \zeta (w_k)P_q \circ CONN_{{\mathbb {G}}}(w_1, w_k) = P_q \circ \zeta (w_1)P_q \circ \zeta (w_k)P_q \circ \zeta (w_1)$ for each $1 \le q \le m$. Taking summation over *k*, we have

12 $$\begin{aligned}&\sum _{k = 2}^{n} P_q \circ \zeta (w_1)P_q \circ \zeta (w_k)P_q \circ CONN_{{\mathbb {G}}}(w_1, w_k) \nonumber \\&\quad = (P_q \circ \zeta (w_1))^2 \sum _{k = 2}^{n}P_q \circ \zeta (w_k), \end{aligned}$$

for each $1 \le q \le m$. Consider $P_q \circ \zeta (w_i)P_q \circ \zeta (w_k)P_q \circ CONN_{{\mathbb {G}}}(w_i, w_k)$ for $i = 2, 3, \ldots , n$ and $k = i + 1$ ($k = 3, 4, \ldots , n$). Since, $i < k$ and for any edge $wz \in E$, $P_q \circ \sigma (wz) = \inf \{P_q \circ \zeta (w), P_q \circ \zeta (z)\}$ for each $1 \le q \le m$. Therefore, $P_q \circ \zeta (w_i)P_q \circ \zeta (w_k)P_q \circ CONN_{{\mathbb {G}}}(w_i, w_k) = P_q \circ \zeta (w_i)P_q \circ \zeta (w_k)P_q \circ \zeta (w_i)$ for each $1 \le q \le m$. Taking summation over *i* and *k*, we have

13 $$\begin{aligned}&\sum _{i = 2}^{n}\sum _{k = i + 1}^{n} P_q \circ \zeta (w_i)P_q \circ \zeta (w_k)P_q \circ CONN_{{\mathbb {G}}}(w_i, w_k) \nonumber \\&\quad = \sum _{i = 2}^{n}(P_q \circ \zeta (w_i))^2 \sum _{k = i + 1}^{n}P_q \circ \zeta (w_k), \end{aligned}$$

for each $1 \le q \le m$. Adding Eqs. (12) and (13) together, we have

$$\begin{aligned}&\sum _{w_i, w_k \in W} P_q \circ \zeta (w_1)P_q \circ \zeta (w_k)P_q \circ CONN_{{\mathbb {G}}}(w_1, w_k)\\&\quad = (P_q \circ \zeta (w_1))^2 \sum _{k = 2}^{n}P_q \circ \zeta (w_k)\\&\qquad + \sum _{i = 2}^{n}(P_q \circ \zeta (w_i))^2 \sum _{k = i + 1}^{n}P_q \circ \zeta (w_k), \end{aligned}$$

for each $1 \le q \le m$. By combining both terms on R.H.S. of above equation, we have

$$\begin{aligned}&P_q \circ CI({\mathbb {G}}) = \sum _{i = 1}^{1}(P_q \circ \zeta (w_i))^2 \\&\quad \sum _{k = i + 1}^{n}P_q \circ \zeta (w_k), \end{aligned}$$

for each $1 \le q \le m$. This completes the proof. $\square$

### Example 7

**Fig. 7:**
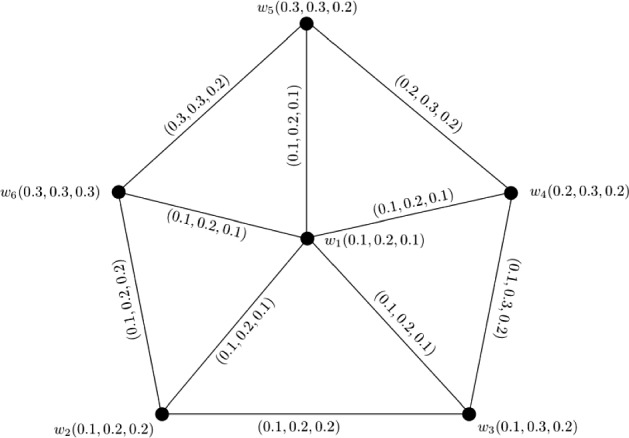
Wheel 3-PF graph ${\mathbb {G}}$

Let $W = \{w_1, w_2, w_3, w_4, w_5, w_6\}$ be a vertex set and $E = \{w_1w_2, w_1w_3, w_1w_4, w_1w_5, w_1w_6, w_2w_3, w_3$ $w_4, w_4w_5, w_5w_6, w_6w_2\}$ be the set of edges. The corresponding 3-PF graph ${\mathbb {G}} = (\zeta , \sigma )$ is given in Fig. 7 with $\zeta (w_1) = (0.1, 0.2, 0.1)$, $\zeta (w_2) = (0.1, 0.2, 0.2)$, $\zeta (w_3) = (0.1, 0.3, 0.2)$, $\zeta (w_4) = (0.2, 0.3, 0.2)$, $\xi(w_5) = (0.3, 0.3, 0.2)$ and $\xi(w_6) = (0.3, 0.3, 0.3)$. Clearly, ${\mathbb {G}}$ is a wheel 3-PF graph satisfying $P_1 \circ \zeta (w_i) \le P_2 \circ \zeta (w_i) \le P_3 \circ \zeta (w_i)$ for $i = 1, 2, \ldots , 6$ and $P_q \circ \zeta (w_1) \le P_q \circ \zeta (w_2) \le \ldots \le P_q \circ \zeta (w_6)$ for each $1 \le q \le 3$.

Therefore, we can calculate $CI({\mathbb {G}})$ by using Theorem 6 as

$$\begin{aligned} CI({\mathbb {G}})= & {} (P_1 \circ CI({\mathbb {G}}), P_2 \circ CI({\mathbb {G}}), P_3 \circ CI({\mathbb {G}}))\\= & {} \left( \sum _{i = 1}^{n}(P_1 \circ \zeta (w_i))^{2}\sum _{k = i + 1}^{n}P_1 \circ \zeta (w_k), \sum _{i = 1}^{n}(P_2 \circ \zeta (w_i))^{2}\sum _{k = i + 1}^{n}P_2 \circ \zeta (w_k),\right. \\&\left. \sum _{i = 1}^{n}(P_3 \circ \zeta (w_i))^{2}\sum _{k = i + 1}^{n}P_3 \circ \zeta (w_k)\right) . \end{aligned}$$

Here,

$$\begin{aligned} P_1 \circ CI({\mathbb {G}})= & {} \sum _{i = 1}^{6}(P_1 \circ \zeta (w_i))^{2}\sum _{k = i + 1}^{6}P_1 \circ \zeta (w_k)\\= & {} (P_1 \circ \zeta (w_1))^{2}\sum _{k = 2}^{6}P_1 \circ \zeta (w_k) + P_1 \circ \zeta (w_2))^{2}\sum _{k = 3}^{6}P_1 \circ \zeta (w_k) \\&+ P_1 \circ \zeta (w_3))^{2}\sum _{k = 4}^{6}P_1 \circ \zeta (w_k)\\&+ P_1 \circ \zeta (w_4))^{2}\sum _{k = 5}^{6}P_1 \circ \zeta (w_k) + P_1 \circ \zeta (w_5))^{2}P_1 \circ \zeta (w_6)\\= & {} (P_1 \circ \zeta (w_1))^{2}P_1 \circ \zeta (w_2) + (P_1 \circ \zeta (w_1))^{2}P_1 \circ \zeta (w_3) \\&+ (P_1 \circ \zeta (w_1))^{2}P_1 \circ \zeta (w_4)\\&+ (P_1 \circ \zeta (w_1))^{2}P_1 \circ \zeta (w_5) + (P_1 \circ \zeta (w_1))^{2}P_1 \circ \zeta (w_6) \\&+ (P_1 \circ \zeta (w_2))^{2}P_1 \circ \zeta (w_3)\\&+ (P_1 \circ \zeta (w_2))^{2}P_1 \circ \zeta (w_4) + (P_1 \circ \zeta (w_2))^{2}P_1 \circ \zeta (w_5) \\&+ (P_1 \circ \zeta (w_2))^{2}P_1 \circ \zeta (w_6)\\&+ (P_1 \circ \zeta (w_3))^{2}P_1 \circ \zeta (w_4) + (P_1 \circ \zeta (w_3))^{2}P_1 \circ \zeta (w_5) \\&+ (P_1 \circ \zeta (w_3))^{2}P_1 \circ \zeta (w_6)\\&+ (P_1 \circ \zeta (w_4))^{2}P_1 \circ \zeta (w_5) + (P_1 \circ \zeta (w_4))^{2}P_1 \circ \zeta (w_6)\\&+ (P_1 \circ \zeta (w_5))^{2}P_1 \circ \zeta (w_6)\\= & {} (0.1)^{2}0.1 + (0.1)^{2}0.1 + (0.1)^{2}0.2 + (0.1)^{2}0.3 + (0.1)^{2}0.3\\&+ (0.1)^{2}0.1 + (0.1)^{2}0.2 + (0.1)^{2}0.3 + (0.1)^{2}0.3 + (0.1)^{2}0.2\\&+ (0.1)^{2}0.3 + (0.1)^{2}0.3 + (0.2)^{2}0.3 + (0.2)^{2}0.3 + (0.3)^{2}0.3\\= & {} 0.01 + 0.009 + 0.008 + 0.024 + 0.027\\= & {} 0.078. \end{aligned}$$

Similarly, we obtain $P_2 \circ CI({\mathbb {G}}) = 0.266$ and $P_3 \circ CI({\mathbb {G}}) = 0.107$. After calculations, we have

$$\begin{aligned} CI({\mathbb {G}})= & {} (0.078, 0.266, 0.107). \end{aligned}$$

## Connectivity index of edge-deleted *m*-PF subgraphs

We observed that the elimination of a vertex *w* from an *m*-PF graph ${\mathbb {G}}$ produces a new *m*-PF subgraph $\widetilde{{\mathbb {G}}} = {\mathbb {G}} \setminus \{w\}$ whose connectivity index is smaller. This was presented as the particular case 1 of Proposition 1. However, the connectivity index of edge-deleted *m*-PF subgraphs of an *m*-PF graph ${\mathbb {G}}$ depends on the nature the deleted edge. It may remain same or reduce. In this section, we examine the connectivity index of edge-deleted *m*-PF subgraphs of an *m*-PF graph. Consider the following examples.

### Example 8

**Fig. 8:**
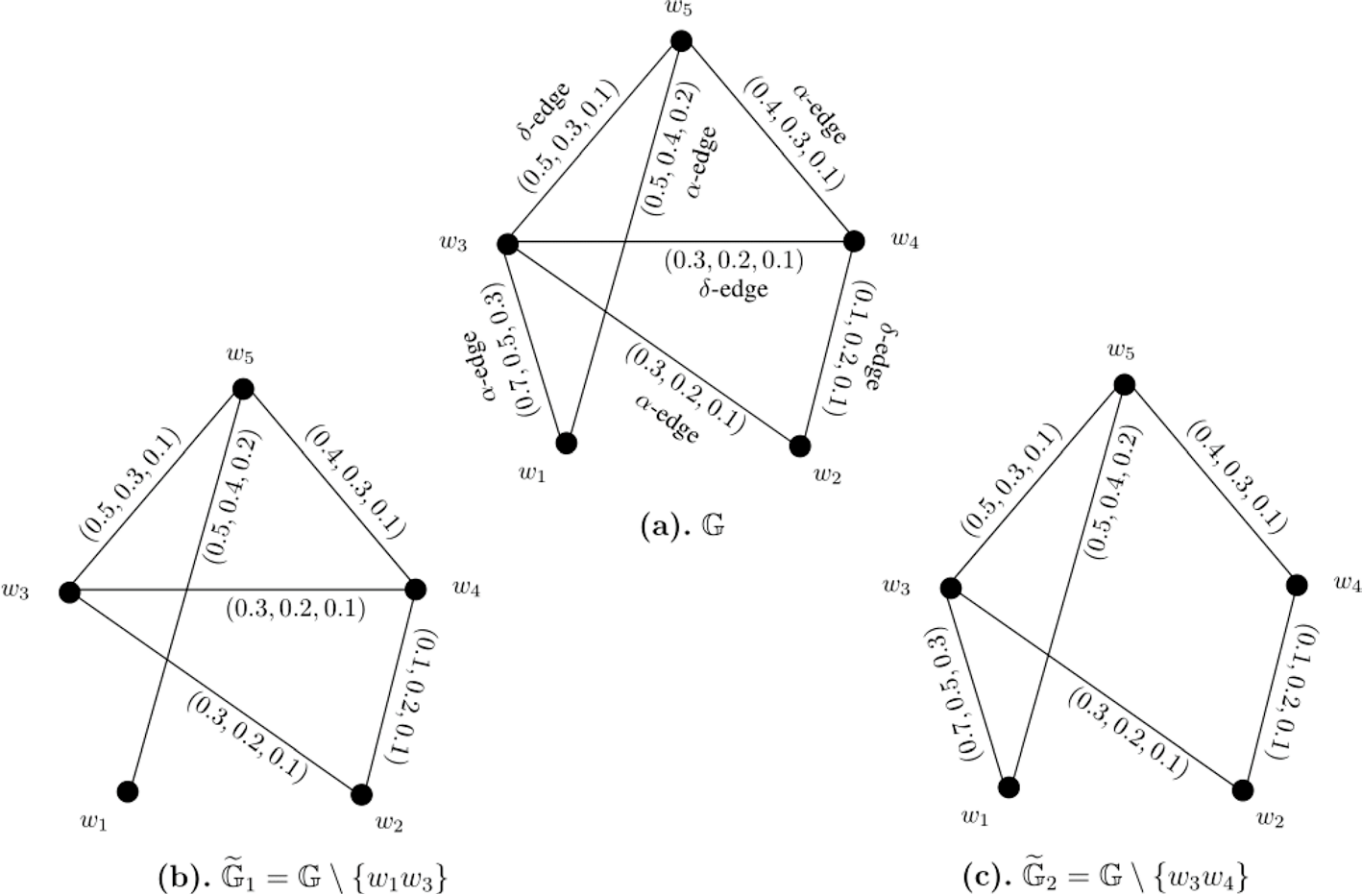
3-PF graph ${\mathbb {G}}$, edge-deleted 3-PF subgraphs $\widetilde{{\mathbb {G}}}_1 = {\mathbb {G}} \setminus \{w_1w_3\}$ and $\widetilde{{\mathbb {G}}}_2 = {\mathbb {G}} \setminus \{w_3w_4\}$ of ${\mathbb {G}}$

Let $W = \{w_1, w_2, w_3, w_4, w_5\}$ be a vertex set and $E = \{w_1w_3, w_1w_5, w_2w_3, w_2w_4, w_3w_4, w_3w_5, w_4w_5\}$ be the set of edges. The corresponding 3-PF graph ${\mathbb {G}} = (\zeta , \sigma )$ is given in Fig. 8a with $\zeta (w_i) = (1, 1, 1)$ for all $i = 1, 2, \ldots , 5$. The strength of connectedness between all pairs of vertices of ${\mathbb {G}}$ are calculated in the following connectivity matrix (14). After calculations, we have $CI({\mathbb {G}}) = (4.1, 3.0, 1.4)$. Now, consider edge-deleted 3-PF subgraphs $\widetilde{{\mathbb {G}}}_1 = {\mathbb {G}} \setminus \{w_1w_3\}$ (given in Fig. 8b) and $\widetilde{{\mathbb {G}}}_2 = {\mathbb {G}} \setminus \{w_3w_4\}$ (given in Fig. 8c) of ${\mathbb {G}}$. The strength of connectedness between all pairs of vertices of $\widetilde{{\mathbb {G}}}_1$ and $\widetilde{{\mathbb {G}}}_2$ are calculated in the connectivity matrices respectively shown by Eqs. (15) and (16). Some computations produce $CI(\widetilde{{\mathbb {G}}}_1) = (3.8, 2.6, 1.1)$ and $CI(\widetilde{{\mathbb {G}}}_2) = (4.1, 3.0, 1.4)$.

14 $$\begin{aligned}&\left[ \begin{array}{ccccc} (0.0, 0.0, 0.0) &{} (0.3, 0.2, 0.1) &{} (0.7, 0.5, 0.3) &{} (0.4, 0.3, 0.1) &{} (0.5, 0.4, 0.2)\\ (0.3, 0.2, 0.1) &{} (0.0, 0.0, 0.0) &{} (0.3, 0.2, 0.1) &{} (0.3, 0.2, 0.1) &{} (0.3, 0.2, 0.1)\\ (0.7, 0.5, 0.3) &{} (0.3, 0.2, 0.1) &{} (0.0, 0.0, 0.0) &{} (0.4, 0.3, 0.1) &{} (0.5, 0.4, 0.2)\\ (0.4, 0.3, 0.1) &{} (0.3, 0.2, 0.1) &{} (0.4, 0.3, 0.1) &{} (0.0, 0.0, 0.0) &{} (0.4, 0.3, 0.1)\\ (0.5, 0.4, 0.2) &{} (0.3, 0.2, 0.1) &{} (0.5, 0.4, 0.2) &{} (0.4, 0.3, 0.1) &{} (0.0, 0.0, 0.0)\\ \end{array} \right] \end{aligned}$$

15 $$\begin{aligned}&\left[ \begin{array}{ccccc} (0.0, 0.0, 0.0) &{} (0.3, 0.2, 0.1) &{} (0.5, 0.3, 0.1) &{} (0.4, 0.3, 0.1) &{} (0.5, 0.4, 0.2)\\ (0.3, 0.2, 0.1) &{} (0.0, 0.0, 0.0) &{} (0.3, 0.2, 0.1) &{} (0.3, 0.2, 0.1) &{} (0.3, 0.2, 0.1)\\ (0.5, 0.3, 0.1) &{} (0.3, 0.2, 0.1) &{} (0.0, 0.0, 0.0) &{} (0.3, 0.2, 0.1) &{} (0.5, 0.3, 0.1)\\ (0.4, 0.3, 0.1) &{} (0.3, 0.2, 0.1) &{} (0.3, 0.2, 0.1) &{} (0.0, 0.0, 0.0) &{} (0.4, 0.3, 0.1)\\ (0.5, 0.4, 0.2) &{} (0.3, 0.2, 0.1) &{} (0.5, 0.3, 0.1) &{} (0.4, 0.3, 0.1) &{} (0.0, 0.0, 0.0)\\ \end{array} \right] \end{aligned}$$

16 $$\begin{aligned}&\left[ \begin{array}{ccccc} (0.0, 0.0, 0.0) &{} (0.3, 0.2, 0.1) &{} (0.7, 0.5, 0.3) &{} (0.4, 0.3, 0.1) &{} (0.5, 0.4, 0.2)\\ (0.3, 0.2, 0.1) &{} (0.0, 0.0, 0.0) &{} (0.3, 0.2, 0.1) &{} (0.3, 0.2, 0.1) &{} (0.3, 0.2, 0.1)\\ (0.7, 0.5, 0.3) &{} (0.3, 0.2, 0.1) &{} (0.0, 0.0, 0.0) &{} (0.4, 0.3, 0.1) &{} (0.5, 0.4, 0.2)\\ (0.4, 0.3, 0.1) &{} (0.3, 0.2, 0.1) &{} (0.4, 0.3, 0.1) &{} (0.0, 0.0, 0.0) &{} (0.4, 0.3, 0.1)\\ (0.5, 0.4, 0.2) &{} (0.3, 0.2, 0.1) &{} (0.5, 0.4, 0.2) &{} (0.4, 0.3, 0.1) &{} (0.0, 0.0, 0.0)\\ \end{array} \right] \end{aligned}$$

### Example 9

**Fig. 9:**
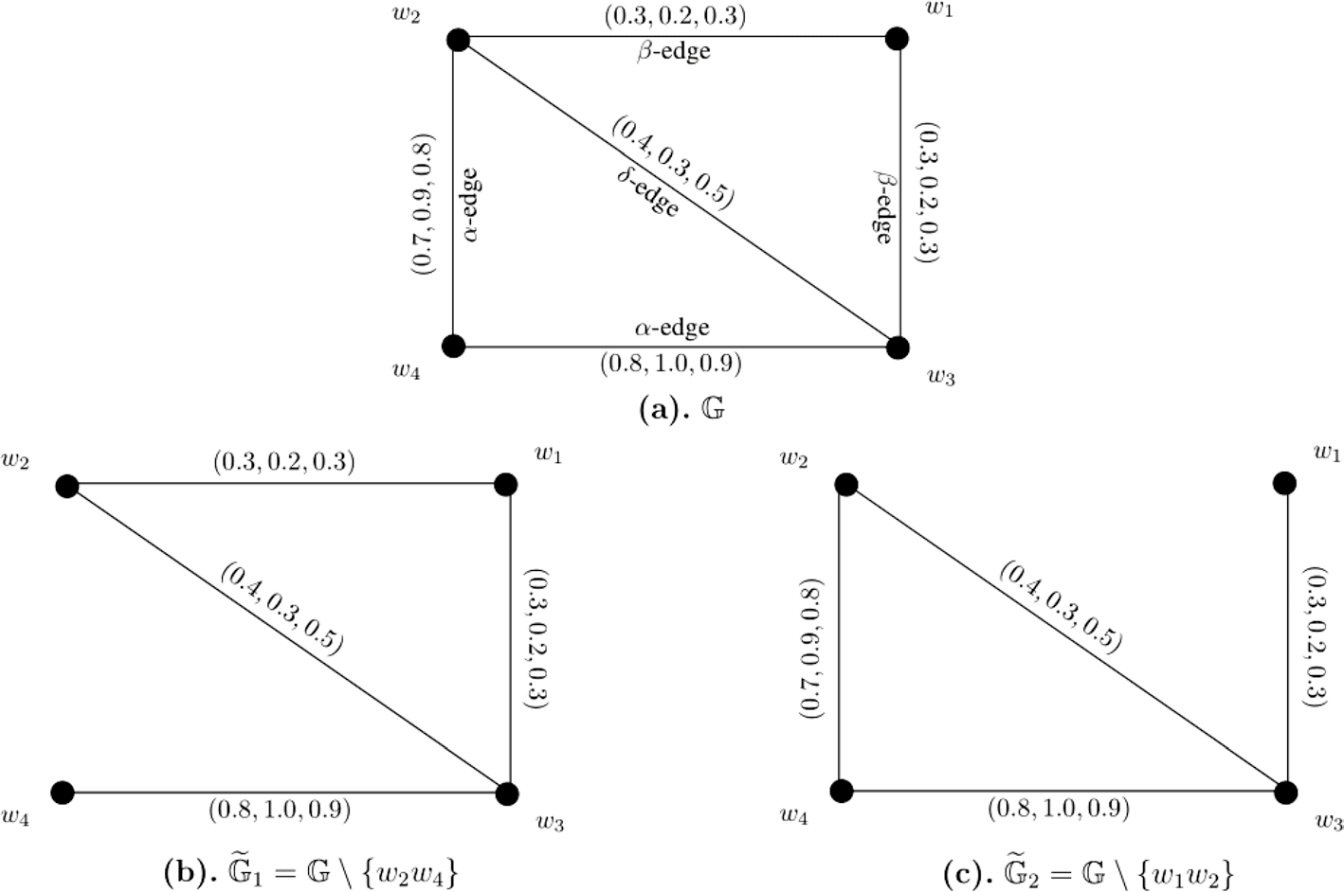
3-PF graph ${\mathbb {G}}$, edge-deleted 3-PF subgraphs $\widetilde{{\mathbb {G}}}_1 = {\mathbb {G}} \setminus \{w_2w_4\}$ and $\widetilde{{\mathbb {G}}}_2 = {\mathbb {G}} \setminus \{w_1w_2\}$ of ${\mathbb {G}}$

Let $W = \{w_1, w_2, w_3, w_4\}$ be a vertex set and $E = \{w_1w_2, w_1w_3, w_2w_3, w_2w_4, w_3w_4\}$ be the set of edges. The corresponding 3-PF graph ${\mathbb {G}} = (\zeta , \sigma )$ is given in Fig. 9a with $\zeta (w_i) = (1, 1, 1)$ for all $i = 1, 2, \ldots , 4$. The strength of connectedness between all pairs of vertices of ${\mathbb {G}}$ are calculated in the following connectivity matrix (17). After calculations, we have $CI({\mathbb {G}}) = (3.1, 3.4, 3.4)$. Now, consider edge-deleted 3-PF subgraphs $\widetilde{{\mathbb {G}}}_1 = {\mathbb {G}} \setminus \{w_2w_4\}$ (given in Fig. 9b) and $\widetilde{{\mathbb {G}}}_2 = {\mathbb {G}} \setminus \{w_1w_2\}$ (given in Fig. 9c) of ${\mathbb {G}}$. The strength of connectedness between all pairs of vertices of $\widetilde{{\mathbb {G}}}_1$ and $\widetilde{{\mathbb {G}}}_2$ are calculated in the connectivity matrices respectively shown in Eqs. (18) and (19). After calculations, we have $CI(\widetilde{{\mathbb {G}}}_1) = (2.5, 2.2, 2.8)$ and $CI(\widetilde{{\mathbb {G}}}_2) = (3.1, 3.4, 3.4)$.

17 $$\begin{aligned}&\left[ \begin{array}{cccc} (0.0, 0.0, 0.0) &{} (0.3, 0.2, 0.3) &{} (0.3, 0.2, 0.3) &{} (0.3, 0.2, 0.3)\\ (0.3, 0.2, 0.3) &{} (0.0, 0.0, 0.0) &{} (0.7, 0.9, 0.8) &{} (0.7, 0.9, 0.8)\\ (0.3, 0.2, 0.3) &{} (0.7, 0.9, 0.8) &{} (0.0, 0.0, 0.0) &{} (0.8, 1.0, 0.9)\\ (0.3, 0.2, 0.3) &{} (0.7, 0.9, 0.8) &{} (0.8, 1.0, 0.9) &{} (0.0, 0.0, 0.0)\\ \end{array} \right] \end{aligned}$$

18 $$\begin{aligned}&\left[ \begin{array}{cccc} (0.0, 0.0, 0.0) &{} (0.3, 0.2, 0.1) &{} (0.7, 0.5, 0.3) &{} (0.4, 0.3, 0.1)\\ (0.3, 0.2, 0.1) &{} (0.0, 0.0, 0.0) &{} (0.4, 0.3, 0.5) &{} (0.4, 0.3, 0.5)\\ (0.7, 0.5, 0.3) &{} (0.4, 0.3, 0.5) &{} (0.0, 0.0, 0.0) &{} (0.4, 0.3, 0.1)\\ (0.4, 0.3, 0.1) &{} (0.4, 0.3, 0.5) &{} (0.4, 0.3, 0.1) &{} (0.0, 0.0, 0.0)\\ \end{array} \right] \end{aligned}$$

19 $$\begin{aligned}&\left[ \begin{array}{cccc} (0.0, 0.0, 0.0) &{} (0.3, 0.2, 0.3) &{} (0.3, 0.2, 0.3) &{} (0.3, 0.2, 0.3)\\ (0.3, 0.2, 0.3) &{} (0.0, 0.0, 0.0) &{} (0.7, 0.9, 0.8) &{} (0.7, 0.9, 0.8)\\ (0.3, 0.2, 0.3) &{} (0.7, 0.9, 0.8) &{} (0.0, 0.0, 0.0) &{} (0.8, 1.0, 0.9)\\ (0.3, 0.2, 0.3) &{} (0.7, 0.9, 0.8) &{} (0.8, 1.0, 0.9) &{} (0.0, 0.0, 0.0)\\ \end{array} \right] \end{aligned}$$

### Example 10

**Fig. 10:**
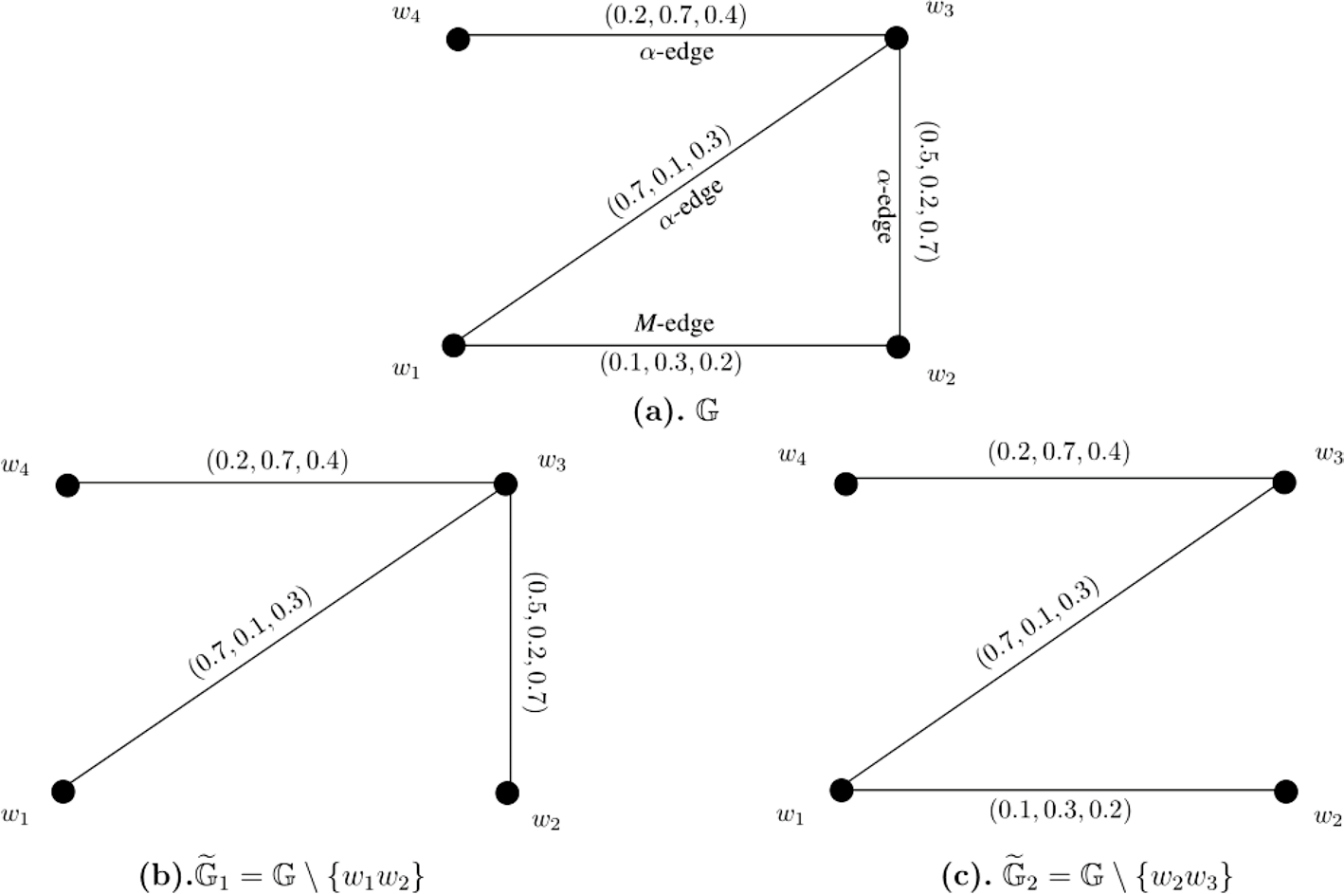
3-PF graph ${\mathbb {G}}$, edge-deleted 3-PF subgraphs $\widetilde{{\mathbb {G}}}_1 = {\mathbb {G}} \setminus \{w_1w_2\}$ and $\widetilde{{\mathbb {G}}}_2 = {\mathbb {G}} \setminus \{w_2w_3\}$ of ${\mathbb {G}}$

Let $W = \{w_1, w_2, w_3, w_4\}$ be a vertex set and $E = \{w_1w_2, w_1w_3, w_2w_3, w_3w_4\}$ be the set of edges. The corresponding 3-PF graph ${\mathbb {G}} = (\zeta , \sigma )$ is given in Fig. 10a with $\zeta (w_i) = (1, 1, 1)$ for all $i = 1, 2, \ldots , 4$. The strength of connectedness between all pairs of vertices of ${\mathbb {G}}$ are calculated in the connectivity matrix shown in Eq. (20). After calculations, we have $CI({\mathbb {G}}) = (2.3, 1.8, 2.4)$. Now, consider edge-deleted 3-PF subgraphs $\widetilde{{\mathbb {G}}}_1 = {\mathbb {G}} \setminus \{w_1w_2\}$ (given in Fig. 10b) and $\widetilde{{\mathbb {G}}}_2 = {\mathbb {G}} \setminus \{w_2w_3\}$ (given in Fig. 10c) of ${\mathbb {G}}$. The strength of connectedness between all pairs of vertices of $\widetilde{{\mathbb {G}}}_1$ and $\widetilde{{\mathbb {G}}}_2$ are calculated in the connectivity matrices respectively shown in Eqs. (21) and (22), respectively. After calculations, we have $CI(\widetilde{{\mathbb {G}}}_1) = (2.3, 1.4, 2.4)$ and $CI(\widetilde{{\mathbb {G}}}_2) = (2.3, 1.8, 2.4)$.

20 $$\begin{aligned}&\left[ \begin{array}{cccc} (0.0, 0.0, 0.0) &{} (0.5, 0.3, 0.3) &{} (0.7, 0.2, 0.3) &{} (0.2, 0.2, 0.3)\\ (0.5, 0.3, 0.3) &{} (0.0, 0.0, 0.0) &{} (0.5, 0.2, 0.7) &{} (0.2, 0.2, 0.4)\\ (0.7, 0.2, 0.3) &{} (0.5, 0.2, 0.7) &{} (0.0, 0.0, 0.0) &{} (0.2, 0.7, 0.4)\\ (0.2, 0.2, 0.3) &{} (0.2, 0.2, 0.4) &{} (0.2, 0.7, 0.4) &{} (0.0, 0.0, 0.0)\\ \end{array} \right] \end{aligned}$$

21 $$\begin{aligned}&\left[ \begin{array}{cccc} (0.0, 0.0, 0.0) &{} (0.5, 0.1, 0.3) &{} (0.7, 0.1, 0.3) &{} (0.2, 0.1, 0.3)\\ (0.5, 0.1, 0.3) &{} (0.0, 0.0, 0.0) &{} (0.5, 0.2, 0.7) &{} (0.2, 0.2, 0.4)\\ (0.7, 0.1, 0.3) &{} (0.5, 0.2, 0.7) &{} (0.0, 0.0, 0.0) &{} (0.2, 0.7, 0.4)\\ (0.2, 0.1, 0.3) &{} (0.2, 0.2, 0.4) &{} (0.2, 0.7, 0.4) &{} (0.0, 0.0, 0.0)\\ \end{array} \right] \end{aligned}$$

22 $$\begin{aligned}&\left[ \begin{array}{cccc} (0.0, 0.0, 0.0) &{} (0.1, 0.3, 0.2) &{} (0.7, 0.1, 0.3) &{} (0.2, 0.1, 0.3)\\ (0.1, 0.3, 0.2) &{} (0.0, 0.0, 0.0) &{} (0.1, 0.1, 0.2) &{} (0.1, 0.1, 0.2)\\ (0.7, 0.1, 0.3) &{} (0.1, 0.1, 0.2) &{} (0.0, 0.0, 0.0) &{} (0.2, 0.7, 0.4)\\ (0.2, 0.1, 0.3) &{} (0.1, 0.1, 0.2) &{} (0.2, 0.7, 0.4) &{} (0.0, 0.0, 0.0)\\ \end{array} \right] \end{aligned}$$

We observed in Example 8, Example 9, and Example 10 that the removal of distinct edges effects the connectivity index of an *m*-PF graph ${\mathbb {G}}$ differently. Therefore, we have several results for characterizing the connectivity index of ${\mathbb {G}}$ as a consequence of this.

### Theorem 7

Let ${\mathbb {G}} = (\zeta , \sigma )$ be an *m*-PF graph and $\widetilde{{\mathbb {G}}} = {\mathbb {G}} \setminus \{wz\}$ be the *m*-PF subgraph of ${\mathbb {G}}$ obtained after deleting an edge $wz \in E$ from ${\mathbb {G}}$. Then for each $1 \le q \le m$, $P_q \circ CI(\widetilde{{\mathbb {G}}}) < P_q \circ CI({\mathbb {G}})$ if and only if *wz* is an *m*-PF bridge of ${\mathbb {G}}$.

### Proof

Let ${\mathbb {G}} = (\zeta , \sigma )$ be an *m*-PF graph and $\widetilde{{\mathbb {G}}} = {\mathbb {G}} \setminus \{wz\}$ be the *m*-PF subgraph of ${\mathbb {G}}$ obtained after deleting an edge $wz \in E$ from ${\mathbb {G}}$. First, suppose that edge *wz* be an *m*-PF bridge of ${\mathbb {G}}$. This implies that for each $1 \le q \le m$, either $P_q \circ CONN_{\widetilde{{\mathbb {G}}}}(u, v) = 0$ or $P_q \circ CONN_{\widetilde{{\mathbb {G}}}}(u, v) < P_q \circ CONN_{{\mathbb {G}}}(u, v)$ for some pair of vertices *u* and *v* of $\widetilde{{\mathbb {G}}}$. This implies that for each $1 \le q \le m$, $P_q \circ \zeta (u) P_q \circ \zeta (v) P_q \circ CONN_{\widetilde{{\mathbb {G}}}}(u, v) < P_q \circ \zeta (u) P_q \circ \zeta (v) P_q \circ CONN_{{\mathbb {G}}}(u, v)$. Taking summation on both sides implies that for each $1 \le q \le m$, $\sum _{u, v \in \widetilde{{\mathbb {G}}}}P_q \circ \zeta (u) P_q \circ \zeta (v) P_q \circ CONN_{\widetilde{{\mathbb {G}}}}(u, v) < \sum _{u, v \in G}P_q \circ \zeta (u) P_q \circ \zeta (v) P_q \circ CONN_{{\mathbb {G}}}(u, v)$. This implies that for each $1 \le q \le m$, $P_q \circ CI(\widetilde{{\mathbb {G}}}) < P_q \circ CI({\mathbb {G}})$. Conversely, suppose that for each $1 \le q \le m$, $P_q \circ CI(\widetilde{{\mathbb {G}}}) < P_q \circ CI({\mathbb {G}})$. To prove that edge yz is an *m*-PF bridge of ${\mathbb {G}}$, consider the following three cases:

**Case 1** Let *wz* be a $\delta$-edge. This means that for each $1 \le q \le m$, $P_q \circ CONN_{\widetilde{{\mathbb {G}}}}(u, v) = P_q \circ CONN_{{\mathbb {G}}}(u, v)$ for all pairs of vertices *u* and *v* of $\widetilde{{\mathbb {G}}}$. This implies that for each $1 \le q \le m$, $P_q \circ \zeta (u) P_q \circ \zeta (v) P_q \circ CONN_{\widetilde{{\mathbb {G}}}}(u, v) = P_q \circ \zeta (u) P_q \circ \zeta (v) P_q \circ CONN_{{\mathbb {G}}}(u, v)$. Taking summation on both sides implies that for each $1 \le q \le m$, $\sum _{u, v \in \widetilde{{\mathbb {G}}}}P_q \circ \zeta (u) P_q \circ \zeta (v) P_q \circ CONN_{\widetilde{{\mathbb {G}}}}(u, v) = \sum _{u, v \in G}P_q \circ \zeta (u) P_q \circ \zeta (v) P_q \circ CONN_{{\mathbb {G}}}(u, v)$. This implies that for each $1 \le q \le m$, $P_q \circ CI(\widetilde{{\mathbb {G}}}) = P_q \circ CI({\mathbb {G}})$. This contradicts to our supposition.

**Case 2** Let *wz* be a $\beta$-edge. This means that for each $1 \le q \le m$, $P_q \circ \sigma (w, z) = P_q \circ CONN_{\widetilde{{\mathbb {G}}}}(w, z)$. This implies that there exists an alternate strongest *m*-PF path between vertices *w* and *z* in ${\mathbb {G}}$ other than the edge *wz* in ${\mathbb {G}}$. This means that the deletion of edge *wz* does not effect the strength of connectedness between any pair of vertices of $\widetilde{{\mathbb {G}}}$ (since, $\widetilde{{\mathbb {G}}}$ is a connected *m*-PF graph). This implies that for each $1 \le q \le m$, $P_q \circ CONN_{\widetilde{{\mathbb {G}}}}(u, v) = P_q \circ CONN_{{\mathbb {G}}}(u, v)$. This shows that for each $1 \le q \le m$, $P_q \circ \zeta (u) P_q \circ \zeta (v) P_q \circ CONN_{\widetilde{{\mathbb {G}}}}(u, v) = P_q \circ \zeta (u) P_q \circ \zeta (v) P_q \circ CONN_{{\mathbb {G}}}(u, v)$. Taking summation on both sides implies that for each $1 \le q \le m$, $\sum _{u, v \in \widetilde{{\mathbb {G}}}}P_q \circ \zeta (u) P_q \circ \zeta (v) P_q \circ CONN_{\widetilde{{\mathbb {G}}}}(u, v) = \sum _{u, v \in G}P_q \circ \zeta (u) P_q \circ \zeta (v) P_q \circ CONN_{{\mathbb {G}}}(u, v)$. This implies that for each $1 \le q \le m$, $P_q \circ CI(\widetilde{{\mathbb {G}}}) = P_q \circ CI({\mathbb {G}})$. This again contradicts to our supposition.

**Case 3** Let *wz* be an $\alpha$-edge. This means that for each $1 \le q \le m$, $P_q \circ \sigma (w, z) > P_q \circ CONN_{\widetilde{{\mathbb {G}}}}(w, z)$. This implies that edge *wz* is the unique strongest *m*-PF path between vertices *w* and *z* in ${\mathbb {G}}$. This means that for each $1 \le q \le m$, $P_q \circ CONN_{\widetilde{{\mathbb {G}}}}(w, z) < P_q \circ CONN_{{\mathbb {G}}}(w, z)$. This implies that for each $1 \le q \le m$, $P_q \circ \zeta (u) P_q \circ \zeta (v) P_q \circ CONN_{\widetilde{{\mathbb {G}}}}(u, v) < P_q \circ \zeta (u) P_q \circ \zeta (v) P_q \circ CONN_{{\mathbb {G}}}(u, v)$. Taking summation on both sides implies that for each $1 \le q \le m$, $\sum _{u, v \in \widetilde{{\mathbb {G}}}}P_q \circ \zeta (u) P_q \circ \zeta (v) P_q \circ CONN_{\widetilde{{\mathbb {G}}}}(u, v) < \sum _{u, v \in G}P_q \circ \zeta (u) P_q \circ \zeta (v) P_q \circ CONN_{{\mathbb {G}}}(u, v)$. This implies that for each $1 \le q \le m$, $P_q \circ CI(\widetilde{{\mathbb {G}}}) < P_q \circ CI({\mathbb {G}})$. Since, $\alpha$-edges are *m*-PF bridges of ${\mathbb {G}}$. Therefore, *wz* is an *m*-PF bridge of ${\mathbb {G}}$. Thus, it is proved that for each $1 \le q \le m$, $P_q \circ CI(\widetilde{{\mathbb {G}}}) < P_q \circ CI({\mathbb {G}})$ if and only if *wz* is an *m*-PF bridge of ${\mathbb {G}}$. $\square$

### Corollary 1

Let ${\mathbb {G}} = (\zeta , \sigma )$ be an *m*-PF graph and $\widetilde{{\mathbb {G}}} = {\mathbb {G}} \setminus \{wz\}$ be the *m*-PF subgraph of ${\mathbb {G}}$. Then for each $1 \le q \le m$, $P_q \circ CI(\widetilde{{\mathbb {G}}}) < P_q \circ CI({\mathbb {G}})$ if and only if *wz* is either $\beta$-edge or $\delta$-edge.

For illustration of Corollary 1, consider Example 8. Since, edge $w_3w_4$ is a $\delta$-edge of ${\mathbb {G}}$, therefore its deletion does not effect the connectivity index of ${\mathbb {G}}$, i.e., $CI(\widetilde{{\mathbb {G}}}_2 = {\mathbb {G}} \setminus \{w_3w_4\}) = (4.1, 3.0, 1.4) = CI({\mathbb {G}})$. Now, consider Example 9. Since, edge $w_1w_2$ is a $\beta$-edge of ${\mathbb {G}}$, therefore its deletion does not effect the connectivity index of ${\mathbb {G}}$, i.e., $CI(\widetilde{{\mathbb {G}}}_2 = {\mathbb {G}} \setminus \{w_1w_2\}) = (3.1, 3.4, 3.4) = CI({\mathbb {G}})$.

### Remark 1

A complete *m*-PF graph ${\mathbb {G}} = (\zeta , \sigma )$ has at most one *m*-PF bridge.

### Example 11

Consider the complete 3-PF graph in Fig. 5 of Example 5. Here, edge $w_3w_4$ is the only $\alpha$-edge of ${\mathbb {G}}$. This means that ${\mathbb {G}}$ has only one 3-PF bridge, namely $w_3w_4$. The rest of the edges are $\beta$-edges.

### Theorem 8

Let ${\mathbb {G}} = (\zeta , \sigma )$ be an *m*-PF graph and $\widetilde{{\mathbb {G}}} = {\mathbb {G}} \setminus \{wz\}$ be the *m*-PF subgraph of ${\mathbb {G}}$. Then for each $1 \le q \le m$, $P_q \circ CI(\widetilde{{\mathbb {G}}}) \ne P_q \circ CI({\mathbb {G}})$ if and only if *wz* is the unique *m*-PF bridge of ${\mathbb {G}}$.

### Proof

Let ${\mathbb {G}} = (\zeta , \sigma )$ be an *m*-PF graph and $\widetilde{{\mathbb {G}}} = {\mathbb {G}} \setminus \{wz\}$ be the *m*-PF subgraph of ${\mathbb {G}}$. First, suppose that for each $1 \le q \le m$, $P_q \circ CI(\widetilde{{\mathbb {G}}}) \ne P_q \circ CI({\mathbb {G}})$. Since, ${\mathbb {G}}$ is a complete *m*-PF graph, therefore by Remark 1, *wz* is the unique *m*-PF bridge of ${\mathbb {G}}$. Conversely, suppose that *wz* is the unique *m*-PF bridge of ${\mathbb {G}}$. Then by Theorem 7, for each $1 \le q \le m$, $P_q \circ CI(\widetilde{{\mathbb {G}}}) < P_q \circ CI({\mathbb {G}})$. This implies that for each $1 \le q \le m$, $P_q \circ CI(\widetilde{{\mathbb {G}}}) \ne P_q \circ CI({\mathbb {G}})$. Thus, it is proved that for each $1 \le q \le m$, $P_q \circ CI(\widetilde{{\mathbb {G}}}) \ne P_q \circ CI({\mathbb {G}})$ if and only if *wz* is the unique *m*-PF bridge of ${\mathbb {G}}$. $\square$

## Average connectivity index of an *m*-PF graph

The only way to assure the stability of a flow in a piece of the network or in the whole network is to measure the average flow in that area. In order to achieve this goal, we present the average connectivity index of *m*-PF graphs in this section. The definition of average connectivity index of an *m*-PF graph ${\mathbb {G}} = (\zeta , \sigma )$ is as follows.

### Definition 14

Let ${\mathbb {G}} = (\zeta , \sigma )$ be an *m*-PF graph. The average connectivity index of ${\mathbb {G}}$ is denoted by $ACI({\mathbb {G}})$ and is defined as $ACI({\mathbb {G}}) = (P_1 \circ ACI({\mathbb {G}}), P_2 \circ ACI({\mathbb {G}}), \ldots , P_m \circ ACI({\mathbb {G}}))$, where $P_1 \circ ACI({\mathbb {G}}) = \frac{1}{n_{C_2}} \sum _{w, z \in W} P_1 \circ \zeta (w) P_1 \circ \zeta (z) P_1 \circ CONN_{{\mathbb {G}}}(w, z)$ for each $1 \le q \le m$. Simply, we can say that an $ACI({\mathbb {G}})$ is obtained by dividing the $CI({\mathbb {G}})$ by total number of pairs of vertices ($n_{C_2}$) of ${\mathbb {G}}$, that is, $ACI({\mathbb {G}}) = \frac{1}{n_{C_2}}[CI({\mathbb {G}})]$. Also, for each $1 \le q \le m$, $0 \le P_q \circ ACI({\mathbb {G}}) \le 1$.

### Example 12

Consider the 3-PF graph ${\mathbb {G}} = (\zeta , \sigma )$ in Fig. 1 of Example 1. Here, $CI({\mathbb {G}}) = (4.6, 3.3, 4.8)$ and $n_{C_2} = 5_{C_2} = 10$. After dividing $CI({\mathbb {G}})$ by 10, we have $ACI({\mathbb {G}}) = (0.46, 0.33, 0.48)$.

We observed in case 1 of Proposition 1 that the deletion of a vertex from an *m*-PF graph ${\mathbb {G}}$ reduces the $CI({\mathbb {G}})$. But how does deleting a vertex from a *m*-PF graph ${\mathbb {G}}$ effect the $ACI({\mathbb {G}})$? To observe the effect look at the following example.

### Example 13

**Fig. 11:**
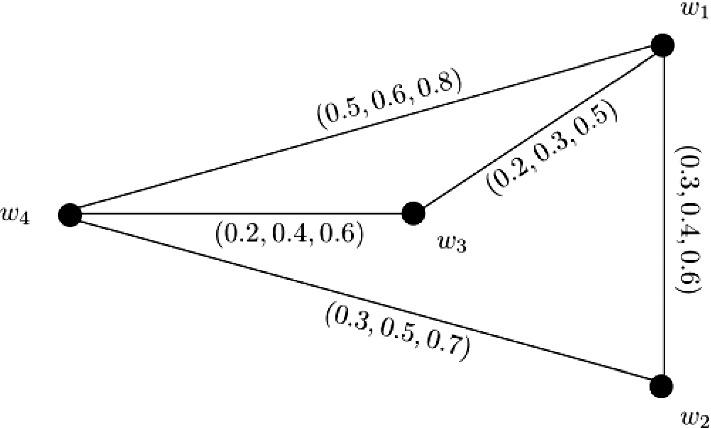
3-PF graph $${\mathbb {G}}$$

Let $W = \{w_1, w_2, w_3, w_4\}$ be a vertex set and $E = \{w_1w_2, w_1w_3, w_1w_4, w_2w_4, w_3w_4\}$ be the set of edges. The corresponding 3-PF graph ${\mathbb {G}} = (\zeta , \sigma )$ is given in Fig. 11 with $\zeta (w_i) = (1, 1, 1)$ for all $i = 1, 2, \ldots , 4$. The strength of connectedness between all pairs of vertices of ${\mathbb {G}}$ are calculated in the following connectivity matrix (23). Here, $n_{C_2} = 4_{C_2} = 6$. After calculations, we have $CI({\mathbb {G}}) = (2.1, 3.1, 3.6)$ and $ACI({\mathbb {G}}) = (0.283, 0.467, 0.667)$. $ACI({\mathbb {G}} \setminus \{w_1\}) = (0.233, 0.433, 0.633)$, $ACI({\mathbb {G}} \setminus \{w_2\}) = (0.300, 0.467, 0.667)$, $ACI({\mathbb {G}} \setminus \{w_3\}) = (0.367, 0.533, 0.733)$, $ACI({\mathbb {G}} \setminus \{w_4\}) = (0.233, 0.333, 0.533)$. Clearly, the deletion of vertices $w_1$ and $w_4$ reduces the $ACI({\mathbb {G}})$ whereas the deletion of vertices $w_2$ and $w_3$ enhances the $ACI({\mathbb {G}})$. The effect of deletion of different vertices on $ACI({\mathbb {G}})$ is shown in Table 2.

23 $$\begin{aligned}&\left[ \begin{array}{cccc} (0.0, 0.0, 0.0) &{} (0.3, 0.5, 0.7) &{} (0.2, 0.4, 0.6) &{} (0.5, 0.6, 0.8)\\ (0.3, 0.5, 0.7) &{} (0.0, 0.0, 0.0) &{} (0.2 0.4, 0.6) &{} (0.3, 0.5, 0.7)\\ (0.2, 0.4, 0.6) &{} (0.2 0.4, 0.6) &{} (0.0, 0.0, 0.0) &{} (0.2, 0.4, 0.6)\\ (0.5, 0.6, 0.8) &{} (0.3, 0.5, 0.7) &{} (0.2, 0.4, 0.6) &{} (0.0, 0.0, 0.0)\\ \end{array} \right] \end{aligned}$$

**Table 2:** Effect on $ACI({\mathbb {G}})$ after deleting a vertex from ${\mathbb {G}}$

${\mathbb {G}} \setminus \{{ w}_{ i}\}$	${ CI}{({\mathbb {G}} \setminus \{{ w}_{ i}\})}$	${ ACI}({\mathbb {G}} \setminus \{{ w}_{ i}\})$	Effect
${\mathbb {G}} \setminus \{{w}_{1}\}$	(0.7, 1.3, 1.9)	(0.233, 0.433, 0.633)	$ACI({\mathbb {G}} \setminus \{w_1\}) < ACI({\mathbb {G}})$
${\mathbb {G}} \setminus \{{w}_{2}\}$	(0.9, 1.4, 2.0)	(0.300, 0.467, 0.67)	$ACI({\mathbb {G}} \setminus \{w_2\}) > ACI({\mathbb {G}})$
${\mathbb {G}} \setminus \{{w}_{3}\}$	(1.1, 1.6, 2.2)	(0.367, 0.533, 0.733)	$ACI({\mathbb {G}} \setminus \{w_3\}) > ACI({\mathbb {G}})$
${\mathbb {G}} \setminus \{{w}_{4}\}$	(0.7, 1.0, 1.6)	(0.233, 0.333, 0.533)	$ACI({\mathbb {G}} \setminus \{w_4\}) < ACI({\mathbb {G}})$

We observed in Example 13 that the deletion of some vertices reduces the average connectivity index of *m*-PF graph, while the deletion of some vertices enhances the average connectivity index of *m*-PF graph. There may be some vertices in *m*-PF graph whose deletion does not effect the its average connectivity index. As a result, we have the following definition for characterizing the vertices of an *m*-PF graph ${\mathbb {G}} = (\zeta , \sigma )$ using the $ACI({\mathbb {G}})$.

### Definition 15

Let ${\mathbb {G}} = (\zeta , \sigma )$ be an *m*-PF graph and $w \in W$. Then *w* is called an *m*-PF connectivity reducing vertex (*m*-PFCRV) or an *m*-PF connectivity enhancing vertex (*m*-PFCEV) or an *m*-PF connectivity neutral vertex (*m*-PFCNV) if for each $1 \le q \le m$, either $P_q \circ ACI({\mathbb {G}} \setminus \{w\}) < P_q \circ ACI({\mathbb {G}})$ or $P_q \circ ACI({\mathbb {G}} \setminus \{w\}) > P_q \circ ACI({\mathbb {G}})$ or $P_q \circ ACI({\mathbb {G}} \setminus \{w\}) = P_q \circ ACI({\mathbb {G}})$, respectively. Otherwise, it is said to be an *m*-PF connectivity mixed vertex (*m*-PFCMV).

Consider the 3-PF graph ${\mathbb {G}} = (\zeta , \sigma )$ in Fig. 11 of Example 13, here $w_1, w_4$ are *m*-PFCRVs and $w_2, w_3$ are *m*-PFCEVs. We now characterize these vertices based on connectivity index of *m*-PFG in the following theorem.

### Theorem 9

Let ${\mathbb {G}} = (\zeta , \sigma )$ be an *m*-PF graph with $|W| = n \ge 3$. Also, let $\frac{P_q \circ CI({\mathbb {G}})}{P_q \circ CI({\mathbb {G}} \setminus \{w\})} = r_q$ for each $1 \le q \le m$ and $w \in W$. Then *w* is said to be an

(i) *m*-PFCRV if and only if $r_q > \frac{n}{n - 2}$ for each $1 \le q \le m$.
(ii) *m*-PFCEV if and only if $r_q < \frac{n}{n - 2}$ for each $1 \le q \le m$.
(iii) *m*-PFCNV if and only if $r_q = \frac{n}{n - 2}$ for each $1 \le q \le m$.

### Proof

Let ${\mathbb {G}} = (\zeta , \sigma )$ be an *m*-PF graph with $|W| = n \ge 3$. Suppose that $\frac{P_q \circ CI({\mathbb {G}})}{P_q \circ CI({\mathbb {G}} \setminus \{w\})} = r_q$ for each $1 \le q \le m$ and $w \in W$. **(i).** Let *w* be an *m*-PFCRV. By Definition 15, $P_q \circ ACI({\mathbb {G}} \setminus \{w\}) < P_q \circ ACI({\mathbb {G}})$ for each $1 \le q \le m$. $\Leftrightarrow \frac{1}{(n - 1)_{C_2}} P_q \circ CI({\mathbb {G}} \setminus \{w\}) < \frac{1}{n_{C_2}} P_q \circ CI({\mathbb {G}})$ for each $1 \le q \le m$. $\Leftrightarrow \frac{P_q \circ CI({\mathbb {G}})}{P_q \circ CI({\mathbb {G}} \setminus \{w\})} < \frac{n_{C_2}}{(n - 1)_{C_2}}$ for each $1 \le q \le m$. $\Leftrightarrow r_q < \frac{n}{n - 2}$ for each $1 \le q \le m$. Similarly, **(ii)** and **(iii)** can be proved. $\square$

### Definition 16

Let ${\mathbb {G}} = (\zeta , \sigma )$ be an *m*-PF graph. Then ${\mathbb {G}}$ is said to be a connectivity reducing *m*-PF graph if it contains at least one *m*-PFCRV. ${\mathbb {G}}$ is said to be a connectivity enhancing *m*-PF graph if it contains at least one *m*-PFCEV. ${\mathbb {G}}$ is said to be a connectivity neutral *m*-PF graph if it contains at least one *m*-PFCNV. Otherwise, it is said to be a connectivity mixed *m*-PF graph.

### Example 14

Consider the 3-PF graph ${\mathbb {G}} = (\zeta , \sigma )$ in Fig. 11 of Example 13. Here, ${\mathbb {G}}$ contains two *m*-PFCRVs, namely $w_1$ and $w_4$, therefore it is a connectivity reducing *m*-PF graph. Also, ${\mathbb {G}}$ contains two *m*-PFCEVs, namely $w_2$ and $w_3$, therefore it is a connectivity enhancing *m*-PF graph. However, ${\mathbb {G}}$ contains no *m*-PFCNV, therefore it is not a connectivity neutral *m*-PF graph.

### Theorem 10

Let ${\mathbb {G}} = (\zeta , \sigma )$ be an *m*-PF graph with $|W| = n \ge 3$. Also, let $\sum _{z \in W \setminus \{w\}} P_q \circ \zeta (w) P_q \circ \zeta (z) P_q \circ CONN_{{\mathbb {G}}}(w, z) = k_q$ for each $1 \le q \le m$ and *w* is an end vertex of ${\mathbb {G}}$. Then *w* is said to be an

(i). *m*-PFCRV if and only if $k_q > \frac{2}{n - 2}$ for each $1 \le q \le m$.
(ii). *m*-PFCEV if and only if $k_q < \frac{2}{n - 2}$ for each $1 \le q \le m$.
(iii). *m*-PFCNV if and only if $k_q = \frac{2}{n - 2}$ for each $1 \le q \le m$.

### Proof

Let ${\mathbb {G}} = (\zeta , \sigma )$ be an *m*-PF graph with $|W| = n \ge 3$. Also, let $\sum _{z \in W \setminus \{w\}} P_q \circ \zeta (w) P_q \circ \zeta (z) P_q \circ CONN_{{\mathbb {G}}}(w, z) = k_q$ for each $1 \le q \le m$ and *w* is an end vertex of ${\mathbb {G}}$. **(i).** First, suppose that *w* be an *m*-PFCRV. Note that $P_q \circ CI({\mathbb {G}}) = P_q \circ CI({\mathbb {G}} \setminus \{w\}) + \sum _{z \in W \setminus \{w\}} P_q \circ \zeta (w) P_q \circ \zeta (z) P_q \circ CONN_{{\mathbb {G}}}(w, z)$ for each $1 \le q \le m$. $\Rightarrow P_q \circ CI({\mathbb {G}}) = P_q \circ CI({\mathbb {G}} \setminus \{w\}) + k_q$ for each $1 \le q \le m$. $\Rightarrow k_q = \frac{P_q \circ CI({\mathbb {G}})}{P_q \circ CI({\mathbb {G}} \setminus \{w\})} - 1$ for each $1 \le q \le m$. Since, *w* is an *m*-PFCRV, therefore by Theorem 9, $k_q > \frac{n}{n - 2} - 1$ for each $1 \le q \le m$. $\Rightarrow k_q > \frac{2}{n - 2}$ for each $1 \le q \le m$. Conversely, suppose that $k_q > \frac{2}{n - 2}$ for each $1 \le q \le m$. Note that $P_q \circ CI({\mathbb {G}}) = P_q \circ CI({\mathbb {G}} \setminus \{w\}) + \sum _{z \in W \setminus \{w\}} P_q \circ \zeta (w) P_q \circ \zeta (z) P_q \circ CONN_{{\mathbb {G}}}(w, z)$ for each $1 \le q \le m$. $\Rightarrow P_q \circ CI({\mathbb {G}}) = P_q \circ CI({\mathbb {G}} \setminus \{w\}) + k_q$ for each $1 \le q \le m$. $\Rightarrow \frac{1}{n_{C_2}} P_q \circ CI({\mathbb {G}}) = \frac{1}{n_{C_2}} P_q \circ CI({\mathbb {G}} \setminus \{w\}) + \frac{1}{n_{C_2}} k_q$ for each $1 \le q \le m$. $\Rightarrow \frac{1}{n_{C_2}} P_q \circ CI({\mathbb {G}}) > \frac{1}{n_{C_2}} P_q \circ CI({\mathbb {G}} \setminus \{w\}) + \frac{1}{n_{C_2}}(\frac{2}{n - 2})$ for each $1 \le q \le m$. By Definition 14, $P_q \circ ACI({\mathbb {G}}) > P_q \circ ACI({\mathbb {G}} \setminus \{w\}) \frac{n - 2}{n} + \frac{1}{n_{C_2}}(\frac{2}{n - 2})$ for each $1 \le q \le m$. $\Rightarrow P_q \circ ACI({\mathbb {G}}) > P_q \circ ACI({\mathbb {G}} \setminus \{w\}) - \frac{2}{n}(P_q \circ ACI({\mathbb {G}} \setminus \{w\}) - \frac{2}{(n - 1)(n - 2)})$ for each $1 \le q \le m$. $\Rightarrow P_q \circ ACI({\mathbb {G}}) > P_q \circ ACI({\mathbb {G}} \setminus \{w\})$ for each $1 \le q \le m$. Hence, *w* is an *m*-PFCRV. Thus, we proved that *w* is an *m*-PFCRV if and only if $k_q > \frac{2}{n - 2}$ for each $1 \le q \le m$. Similarly, **(ii)** and **(iii)** can be proved. $\square$

Below is a general description of the algorithm for calculating different connectivity indices of *m*-PF networks. Its pseudocode presentation is provided in Algorithm 1.

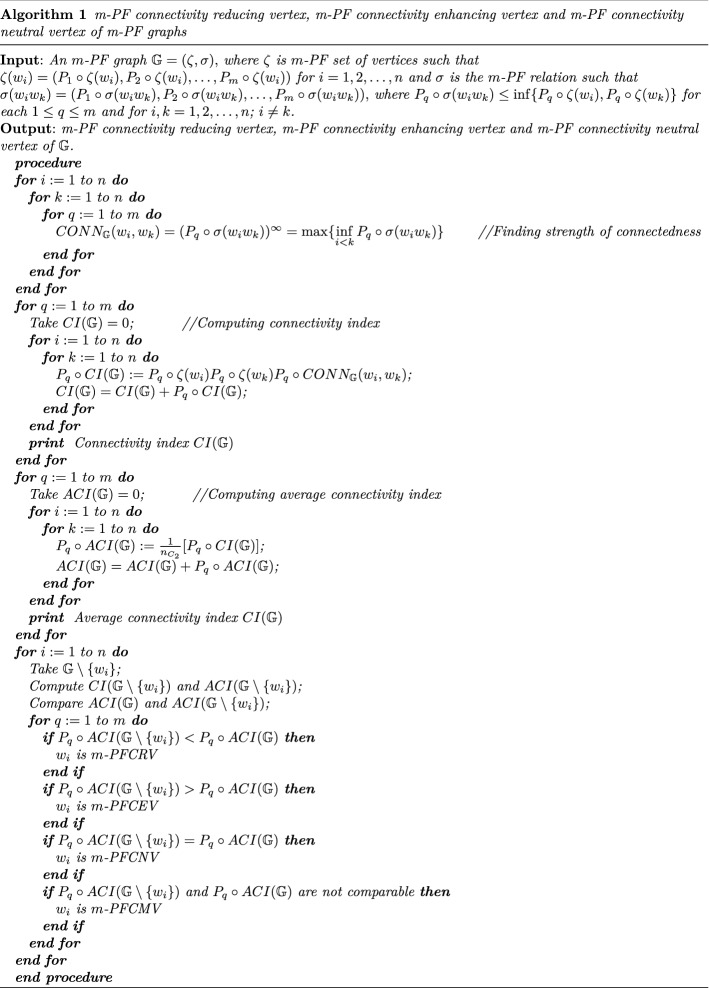



*Description and Complexity of the Algorithm* At the initial stage, the algorithm calculates the strength of connectedness between all pair of nodes, therefore, the time complexity of these nested ‘for’ loops is $O(n^2m),$ where *n* is the total number of nodes in ${\mathbb {G}}.$ The next set of nested loops computes connectivity index of *m*-PF graphs. Its time complexity is $O(mn^2).$ The next stage involves the evaluation of the average connectivity index of *m*-PF graphs. The running time of these nested loops is the same as previous one. The next for loop runs *m* times, therefore its complexity is *O*(*m*). The comparison of different connectivity indices for *m*-PF graphs is then calculated. The running time of all these ‘if’ conditionals is *O*(*m*). This comparison helps to choose more preferable connectivity index. As soon as the connectivity index for a given *m*-PF graph is evaluated, the algorithm will halt. Thus the total time complexity of the algorithm is $O(n^3).$

## Application

One of the most rapidly expanding branches of advanced mathematics is graph theory. It has grown tremendously due to a vast range of applications in optimization problems, combinatorial problems, linguistics, chemistry, physics, biology etc. Connectivity is among the highest priority notions utilized in graph theory applications. In this section, we describe a decision-making process through an application of *m*-PF graphs.

### *m*-PF graphs in product manufacturing problem

Some products may increase the profit of a company if they are offered in multiple places. Before manufacturing a product, every company considers the following important factors.

- Does the product follow the mass market demands?
- Is the product fast or time consuming to manufacture?
- Is the product sold at a high or low cost?
- Does the product appeal the people at global level?

Commonly, graphical models are utilized to solve this type of decision-making issues. *m*-PF graphs are usually adopted in decision-making issues when it is needed to collect a set of individuals. Consider a multinational enterprize (MNE) take a decision to manufacture as few products as possible having more demand, consuming minimum time, attracting a wide range of all classes of people, minimizing cost and giving more profit to the company as compared to the other products. Let MNE consider seven products Prod-I, Prod-II, Prod-II, Prod-IV, Prod-V, Prod-VI and Prod-VII to market them in different countries for earning profit, lowering cost etc. Let the set of products is $W = \{w_1 :$ Prod-I, $w_2 :$ Prod-II, $w_3 :$ Prod-II, $w_4 :$ Prod-IV, $w_5 :$ Prod-V, $w_6 :$ Prod-VI, $w_7 :$ Prod-VII $\}$. This procedure can be described by a 4-PF graph, taking *W* as a vertex set. The membership value of each product illustrates the degree of demand, sale price, time consumption and attraction to people at a global level. The description of the products can be expressed as in the following set:

$\{$Demand, Time, Cost, Appealing$\}$.

Let $\zeta (w_1) = (0.3, 0.2, 0.5, 0.6)$. This means that Prod-I follows $30 \%$ of the mass market, consumes $20 \%$ time to manufacture, $50 \%$ costly or it sales at $50 \%$ high cost and attracts $60 \%$ people at the global level. Similarly, the membership values of the other products are $\zeta (w_2) = (0.3, 0.4, 0.5, 0.7)$, $\zeta (w_3) = (0.5, 0.4, 0.6, 0.7)$, $\zeta (w_4) = (0.1, 0.1, 0.3, 0.4)$, $\zeta (w_5) = (0.1, 0.2, 0.3, 0.5)$, $\zeta (w_6) = (0.2, 0.2, 0.5, 0.6)$ and $\zeta (w_7) = (0.3, 0.3, 0.5, 0.7)$. The edge between two products represents the degree of using common materials, power equipments, engineer employs and agencies involved for both of the products. The description of the pairs of products can be expressed as in the following set:

$\{$Equipments, Materials, Engineer employs, Agencies$\}$.

**Fig. 12:**
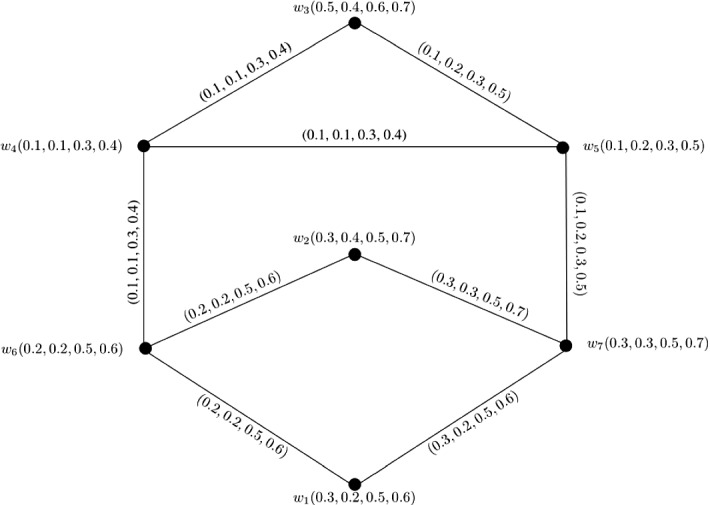
4-PF graph $${\mathbb {G}}$$

Let $\sigma (w_1w_6) = (0.2, 0.2, 0.5, 0.6)$. This means that Prod-I and Prod-VI use $20 \%$ common equipments, $20 \%$ same materials, $50 \%$ common trained engineers and $60 \%$ same agencies. Similarly, the membership values of the other pairs of products are shown in Fig. 12. It can be easily verified that ${\mathbb {G}} = (\zeta , \sigma )$ is a 4-PF graph as shown in Fig. 12. The strength of connectivity between pairs of vertices of ${\mathbb {G}}$ are calculated in Table 3.

**Table 3:** Strength of connectivity between pairs of vertices of ${\mathbb {G}}$

Pairs of vertices	${CONN}_{{\mathbb {G}}}{(w, z)}$	Pairs of vertices	${CONN}_{{\mathbb {G}}}{(w, z)}$
${w, z }\in { W}$		${w, z }\in { W}$	
$w_1, w_2$	(0.3, 0.2, 0.5, 0.6)	$w_3, w_4$	(0.1, 0.1, 0.3, 0.4)
$w_1, w_3$	(0.1, 0.2, 0.3, 0.5)	$w_3, w_5$	(0.1, 0.2, 0.3, 0.5)
$w_1, w_4$	(0.1, 0.1, 0.3, 0.4)	$w_3, w_6$	(0.1, 0.2, 0.3, 0.5)
$w_1, w_5$	(0.1, 0.2, 0.3, 0.5)	$w_3, w_7$	(0.1, 0.2, 0.3, 0.5)
$w_1, w_6$	(0.2, 0.2, 0.5, 0.6)	$w_4, w_5$	(0.1, 0.1, 0.3, 0.4)
$w_1, w_7$	(0.3, 0.2, 0.5, 0.6)	$w_4, w_6$	(0.1, 0.1, 0.3, 0.4)
$w_2, w_3$	(0.1, 0.2, 0.3, 0.5)	$w_4, w_7$	(0.1, 0.1, 0.3, 0.4)
$w_2, w_4$	(0.1, 0.1, 0.3, 0.4)	$w_5, w_6$	(0.1, 0.2, 0.3, 0.5)
$w_2, w_5$	(0.1, 0.2, 0.3, 0.5)	$w_5, w_7$	(0.1, 0.2, 0.3, 0.5)
$w_2, w_6$	(0.2, 0.2, 0.5, 0.6)	$w_6, w_7$	(0.2, 0.2, 0.5, 0.6)
$w_2, w_7$	(0.3, 0.3, 0.5, 0.7)		

**Fig. 13:**
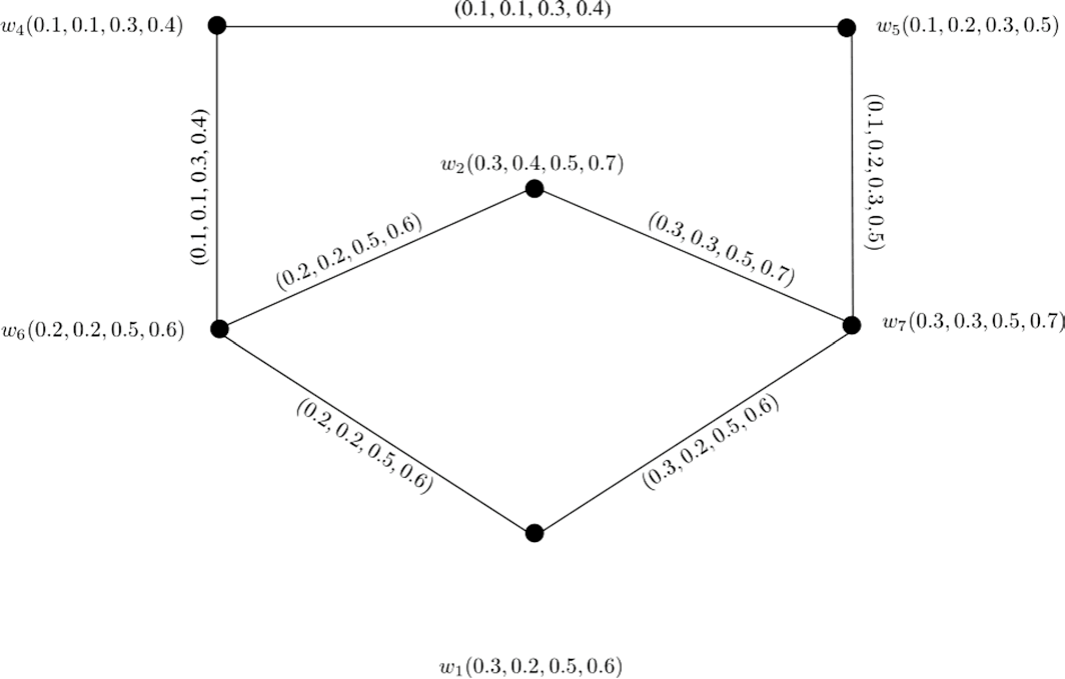
4-PF graph $${\mathbb {G}} \setminus \{w_3\}$$

Here, $n_{C_2} = 7_{C_2} = 21$. After calculations, we have $CI({\mathbb {G}}) = (0.205, 0.373, 1.605, 3.910)$ and $ACI({\mathbb {G}}) = (0.010, 0.018, 0.076, 0.186)$. Removal of certain vertices from ${\mathbb {G}}$ have certain effects on $ACI({\mathbb {G}})$. Consider ${\mathbb {G}} \setminus \{w_3\}$ (see Fig. 13). The strength of connectivity between pairs of vertices of ${\mathbb {G}} \setminus \{w_3\}$ are calculated in Table 4.

**Table 4:** Strength of connectivity between pairs of vertices of ${\mathbb {G}} \setminus \{w_3\}$

Pairs of vertices	${CONN}_{{\mathbb {G}}}{(w, z)}$	Pairs of vertices	${CONN}_{{\mathbb {G}}}{(w, z)}$
${w, z }\in {W }\setminus \{{w}_{3}\}$		${w, z }\in {W} \setminus \{{w}_{3}\}$	
$w_1, w_2$	(0.3, 0.2, 0.5, 0.6)	$w_2, w_7$	(0.3, 0.3, 0.5, 0.7)
$w_1, w_4$	(0.1, 0.1, 0.3, 0.4)	$w_4, w_5$	(0.1, 0.1, 0.3, 0.4)
$w_1, w_5$	(0.1, 0.2, 0.3, 0.5)	$w_4, w_6$	(0.1, 0.1, 0.3, 0.4)
$w_1, w_6$	(0.2, 0.2, 0.5, 0.6)	$w_4, w_7$	(0.1, 0.1, 0.3, 0.4)
$w_1, w_7$	(0.3, 0.2, 0.5, 0.6)	$w_5, w_6$	(0.1, 0.2, 0.3, 0.5)
$w_2, w_4$	(0.1, 0.1, 0.3, 0.4)	$w_5, w_7$	(0.1, 0.2, 0.3, 0.5)
$w_2, w_5$	(0.1, 0.2, 0.3, 0.5)	$w_6, w_7$	(0.2, 0.2, 0.5, 0.6)
$w_2, w_6$	(0.2, 0.2, 0.5, 0.6)		

**Fig. 14:**
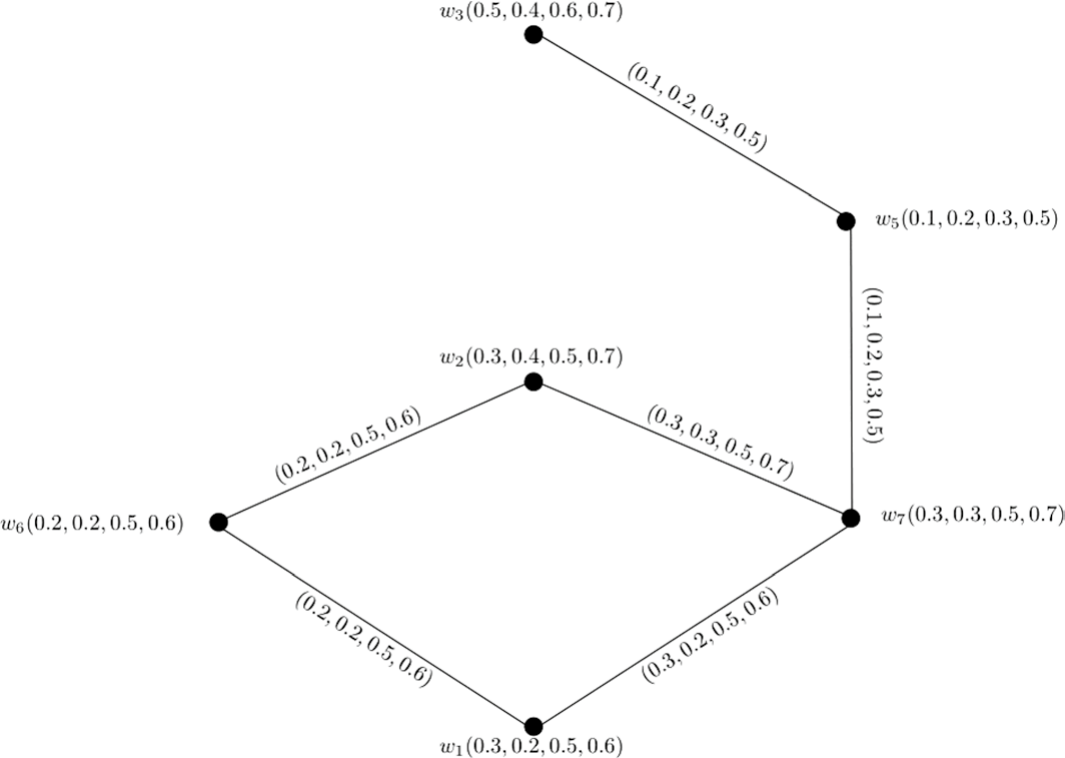
4-PF graph $${\mathbb {G}} \setminus \{w_4\}$$

After calculations, we have $CI({\mathbb {G}} \setminus \{w_3\}) = (0.140, 0.265, 1.137, 2.713)$ and $ACI({\mathbb {G}} \setminus \{w_3\}) = (0.009, 0.018, 0.076,$ 0.181). This means that $ACI({\mathbb {G}} \setminus \{w_3\}) < ACI({\mathbb {G}})$. Now, consider ${\mathbb {G}} \setminus \{w_4\}$ (see Fig. 14). The strength of connectivity between pairs of vertices of ${\mathbb {G}} \setminus \{w_4\}$ are calculated in Table 5.

**Table 5:** Strength of connectivity between pairs of vertices of ${\mathbb {G}} \setminus \{w_4\}$

Pairs of vertices	${CONN}_{{\mathbb {G}}}{(w, z)}$	Pairs of vertices	${CONN}_{{\mathbb {G}}}{(w, z)}$
${w, z }\in {W} \setminus \{{w}_{4}\}$		${w, z }\in {W} \setminus \{{w}_{4}\}$	
$w_1, w_2$	(0.3, 0.2, 0.5, 0.6)	$w_2, w_7$	(0.3, 0.3, 0.5, 0.7)
$w_1, w_3$	(0.1, 0.2, 0.3, 0.5)	$w_3, w_5$	(0.1, 0.2, 0.3, 0.5)
$w_1, w_5$	(0.1, 0.2, 0.3, 0.5)	$w_3, w_6$	(0.1, 0.2, 0.3, 0.5)
$w_1, w_6$	(0.2, 0.2, 0.5, 0.6)	$w_3, w_7$	(0.1, 0.2, 0.3, 0.5)
$w_1, w_7$	(0.3, 0.2, 0.5, 0.6)	$w_5, w_6$	(0.1, 0.2, 0.3, 0.5)
$w_2, w_3$	(0.1, 0.2, 0.3, 0.5)	$w_5, w_7$	(0.1, 0.2, 0.3, 0.5)
$w_2, w_5$	(0.1, 0.2, 0.3, 0.5)	$w_6, w_7$	(0.2, 0.2, 0.5, 0.6)
$w_2, w_6$	(0.2, 0.2, 0.5, 0.6)		

After calculations, we have $CI({\mathbb {G}} \setminus \{w_4\}) = (0.188, 0.356, 1.344, 3.302)$ and $ACI({\mathbb {G}} \setminus \{w_4\}) = (0.013, 0.024,$ 0.090, 0.220). This means that $ACI({\mathbb {G}} \setminus \{w_4\}) > ACI({\mathbb {G}})$. Similarly, the effects of deletion of different vertices on $ACI({\mathbb {G}})$ are shown in Table 6.

**Table 6:** Effect on $ACI({\mathbb {G}})$ after deleting a vertex from ${\mathbb {G}}$

${ {\mathbb {G}} \setminus \{{w}_{i}\}}$	${CI}({\mathbb {G}} \setminus \{{w}_{i}\})$	${ACI}({\mathbb {G}} \setminus \{{w}_{i}\})$	Effect
${ {\mathbb {G}} \setminus \{{w}_{1}\}}$	(0.118, 0.311, 1.050, 2.734)	(0.008, 0.021, 0.070, 0.182)	Mixed
${ {\mathbb {G}} \setminus \{{w}_{2}\}}$	(0.118, 0.253, 1.050, 2.531)	(0.008, 0.017, 0.070, 0.169)	$ACI({\mathbb {G}} \setminus \{w_2\}) < ACI({\mathbb {G}})$
${ {\mathbb {G}} \setminus \{{w}_{3}\}}$	(0.140, 0.265, 1.137, 2.713)	(0.009, 0.018, 0.076, 0.181)	$ACI({\mathbb {G}} \setminus \{w_3\}) < ACI({\mathbb {G}})$
${ {\mathbb {G}} \setminus \{{w}_{4}\}}$	(0.188, 0.356, 1.344, 3.302)	(0.013, 0.024, 0.090, 0.220)	$ACI({\mathbb {G}} \setminus \{w_4\}) > ACI({\mathbb {G}})$
${ {\mathbb {G}} \setminus \{{w}_{5}\}}$	(0.118, 0.244, 1.344, 2.823)	(0.013, 0.016, 0.090, 0.188)	Mixed
${ {\mathbb {G}} \setminus \{{w}_{6}\}}$	(0.154, 0.307, 1.050, 2.709)	(0.010, 0.020, 0.070, 0.181)	Mixed
${ {\mathbb {G}} \setminus \{{w}_{7}\}}$	(0.109, 0.118, 1.050, 2.303)	(0.007, 0.008, 0.070, 0.154)	$ACI({\mathbb {G}} \setminus \{w_7\}) < ACI({\mathbb {G}})$

**Fig. 15:**
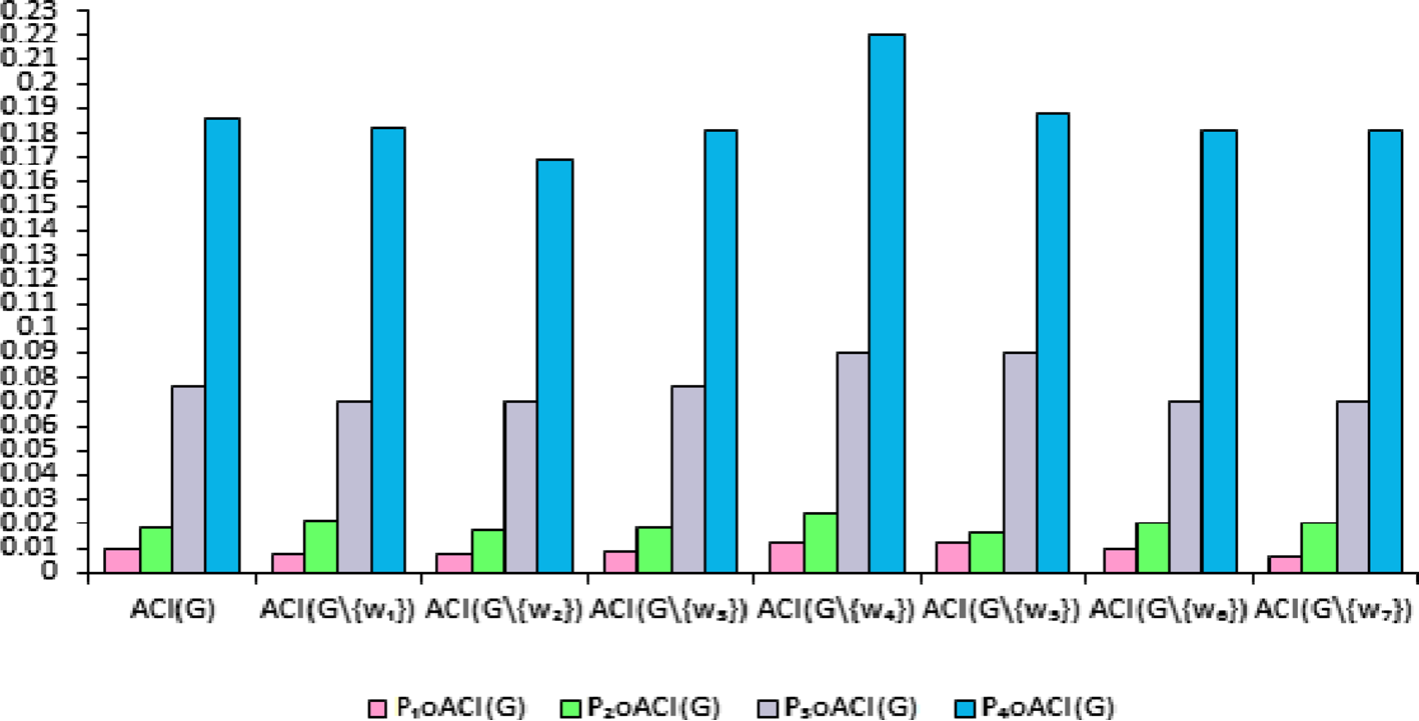
Comparison of $ACI({\mathbb {G}})$ and $ACI({\mathbb {G}} \setminus \{w_i\})$ for $i = 1, 2, \ldots , 7$.

Clearly, the vertices $w_2$, $w_3$, $w_7$ are 4-PFCRVs, vertex $w_4$ is 4-PFCEV and vertices $w_1$, $w_5$, $w_6$ are 4-PFCMVs. Note that if company do not manufacture any one of the products $w_2$, $w_3$ and $w_7$ then its profit will decrease, if company do not manufacture product $w_4$ then its profit will increase and if company do not manufacture any one of the products $w_1$, $w_5$, $w_6$ then there will be mixed change in its profit. Thus, it is sufficient to manufacture the products Prod-*II*, Prod-*III* and Prod-*VII* for minimizing time consumption, earning more profit, attracting a wide range of all classes of people and minimizing cost. The comparison between $ACI({\mathbb {G}})$ and $ACI({\mathbb {G}} \setminus \{w_i\})$ for $i = 1, 2, \ldots , 7$ is analyzed using the bar charts in Fig. 15.

We now describe the general procedure adopted in our application.

Step 1. Input the set of vertices (products) $w_1, w_2, \ldots , w_n$.
Step 2. Input the *m*-PF set $\zeta$ of vertices such that $\zeta (w_i) = (P_1 \circ \zeta (w_i), P_2 \circ \zeta (w_i), \ldots , P_m \circ \zeta (w_i))$
Step 3. Input the set of edges (relationship between products) $w_iw_k$ for $i, k = 1, 2, \ldots , n$; $i \ne k$.
Step 4. Input the *m*-PF relation $\sigma$ of vertices such that $\sigma (w_iw_k) = (P_1 \circ \sigma (w_iw_k), P_2 \circ \sigma (w_iw_k), \ldots , P_m \circ \sigma (w_iw_k))$, where
  $$\begin{aligned}&P_q \circ \sigma (w_iw_k) \le \inf \{P_q \circ \zeta (w_i), P_q \circ \zeta (w_k)\}, \end{aligned}$$
  for each $1 \le q \le m$ and for $i, k = 1, 2, \ldots , n$; $i \ne k$.
Step 5. Construct the *m*-PF graph ${\mathbb {G}} = (\zeta , \sigma )$.
Step 6. Find the strength of connectedness between all pairs of vertices $w_i$ and $w_k$ of ${\mathbb {G}}$ such that $CONN_{{\mathbb {G}}}(w_i, w_k) = ((P_1 \circ \sigma (w_iw_{k}))^{\infty }, (P_2 \circ \sigma (w_iw_{k}))^{\infty }, \ldots , (P_m \circ \sigma (w_iw_{k}))^{\infty })$, where
  $$\begin{aligned} (P_q \circ \sigma (w_i,w_{k}))^{\infty } = \max \{\inf \limits _{1 \le i < k \le n} P_q \circ \sigma (w_iw_{k})\}, \end{aligned}$$
  for each $$1 \le q \le m$$.
Step 7. Compute the $CI({\mathbb {G}}) = (P_1 \circ CI({\mathbb {G}}), P_2 \circ CI({\mathbb {G}}), \ldots , P_m \circ CI({\mathbb {G}}))$ such that
  $$\begin{aligned}&P_q \circ CI({\mathbb {G}}) = \sum _{w_i, w_k \in W}P_q \circ \zeta (w_i)P_q \circ \zeta (w_k)P_q \circ CONN_{{\mathbb {G}}}(w_i, w_k), \end{aligned}$$
  for each $$1 \le q \le m$$.
Step 8. Compute the $ACI({\mathbb {G}}) = (P_1 \circ ACI({\mathbb {G}}), P_2 \circ ACI({\mathbb {G}}), \ldots , P_m \circ ACI({\mathbb {G}}))$ such that
  $$\begin{aligned}&P_q \circ ACI({\mathbb {G}}) = \frac{1}{n_{C_2}}[P_q \circ CI({\mathbb {G}})], \end{aligned}$$
  for each $$1 \le q \le m$$.
Step 9. Consider the *m*-PF subgraph ${\mathbb {G}} \setminus \{w_i\}$ of ${\mathbb {G}}$ by deleting a vertex $w_i$ from ${\mathbb {G}}$.
Step 10. Compute the $CI({\mathbb {G}} \setminus \{w_i\})$ and $ACI({\mathbb {G}} \setminus \{w_i\})$.
Step 11. Compare the $ACI({\mathbb {G}})$ and $ACI({\mathbb {G}} \setminus \{w_i\})$.
Step 12. Output :
  - If $P_q \circ ACI({\mathbb {G}} \setminus \{w_i\}) < P_q \circ ACI({\mathbb {G}})$ for each $1 \le q \le m$ then $w_i$ is *m*-PFCRV.
  - If $P_q \circ ACI({\mathbb {G}} \setminus \{w_i\}) > P_q \circ ACI({\mathbb {G}})$ for each $1 \le q \le m$ then $w_i$ is *m*-PFCEV.
  - If $P_q \circ ACI({\mathbb {G}} \setminus \{w_i\}) = P_q \circ ACI({\mathbb {G}})$ for each $1 \le q \le m$ then $w_i$ is *m*-PFCNV.
  If there is no such comparison as mentioned above between $P_q \circ ACI({\mathbb {G}})$ and $P_q \circ ACI({\mathbb {G}})$ for each $1 \le q \le m$ then $w_i$ is *m*-PFCMV.
Step 13. Repeat steps 9-12 for all vertices $w_1, w_2, \ldots , w_n$.
Step 14. *m*-PFCRVs are preferable here to manufacture by ignoring the other vertices.

## Comparative analysis

*m*-PF graphs have numerous applications in decision-making issues when it is compulsory to make decisions with a group of individuals or agreements. The membership value of an element in an *m*-PF set belongs to $[0, 1]^m$, which exhibits all the *m* different qualities of the object. This is better suited to a variety of real-world uncertain issues when data originates from several agents, resulting in multi-polar information that fuzzy sets and bipolar fuzzy sets cannot accurately express. In this section, we discuss the problem of selecting the set of representatives for a youth development council (YDC) in a university using the fuzzy graph model, bipolar fuzzy graph model and *m*-PF graph model to demonstrate the flexibility and validity of our suggested approach.

### Finding the set of representatives through fuzzy graph

**Fig. 16:**
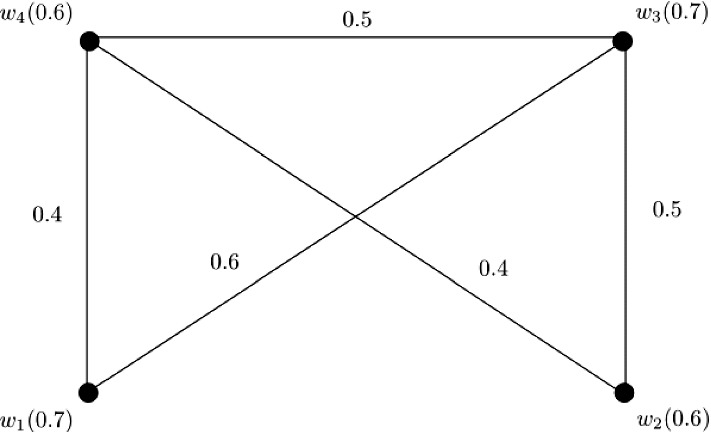
Fuzzy graph $${\mathbb {G}} = (\zeta , \sigma )$$

We consider a set of students $$W = \{w_1 :$$ Hamza, $$w_2 :$$ Suleman, $$w_3 :$$ Waris, $$w_4 :$$ Uzaifa$$\}$$ who want to become a member of YDC. We wish to form a YDC with the fewest possible members. We want to build a YDC in which every member who is not in the council has something in common with those who are. Each student has some good leadership qualities such as approachable, good communicator, good listener, honest and fair. All these qualities are uncertain in nature. Therefore, fuzziness can be added to represent this problem. A fuzzy graph model of this problem is given in Fig. 16, taking *W* as the set of vertices. The membership value of each student represents the degree of having good leadership qualities. For example, $$\zeta ($$Hamza$$) = 0.7$$ means that Hamza has $$70 \%$$ good leadership qualities. The edge between two students represents the degree of having common good leadership qualities. For example, $$\sigma ($$Hamza, Waris$$) = 0.6$$ means that Hamza and Waris have $$60 \%$$ good leadership qualities in common.

The strength of connectivity between pairs of vertices of fuzzy graph ${\mathbb {G}} = (\zeta , \sigma )$ are calculated in Table 7.

**Table 7:** Strength of connectivity between pairs of vertices of ${\mathbb {G}}$

Pairs of vertices	${CONN}_{{\mathbb {G}}}{(w, z)}$
$${w, z }\in { W}$$	
$w_1, w_2$	0.5
$w_1, w_3$	0.6
$w_1, w_4$	0.5
$w_2, w_3$	0.5
$w_2, w_4$	0.5
$w_3, w_4$	0.5

**Fig. 17:**
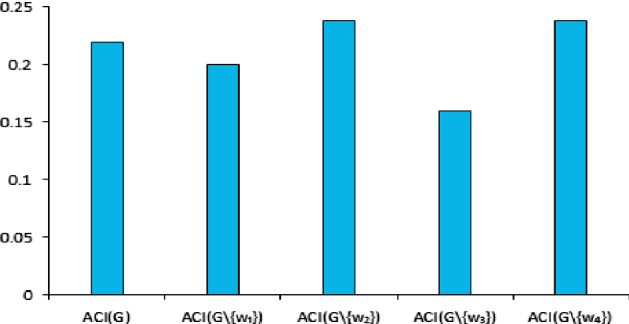
Comparison of $ACI({\mathbb {G}})$ and $ACI({\mathbb {G}} \setminus \{w_i\})$ for $i = 1, 2, 3, 4$

After calculations, we have $CI({\mathbb {G}}) = 1.314$ and $ACI({\mathbb {G}}) = 0.219$. The $CI({\mathbb {G}} \setminus \{w_i\})$ and $ACI({\mathbb {G}} \setminus \{w_i\})$ for $i = 1, 2, 3, 4$ are calculated in Table 8. The effects of elimination of different vertices on $ACI({\mathbb {G}})$ are shown in Fig. 17.

**Table 8:** $ACI({\mathbb {G}})$ after deleting a vertex from ${\mathbb {G}}$

${{\mathbb {G}} \setminus \{{w}_{i}\}}$	${CI}({\mathbb {G}} \setminus \{{w}_{i}\})$	${ACI}({\mathbb {G}} \setminus \{{w}_{i}\})$
${{\mathbb {G}} \setminus \{w_1\}}$	0.600	0.200
${{\mathbb {G}} \setminus \{w_2\}}$	0.714	0.238
${{\mathbb {G}} \setminus \{w_3\}}$	0.480	0.160
${{\mathbb {G}} \setminus \{w_4\}}$	0.714	0.238

Therefore, $w_1$ and $w_3$ should be the members of YDC.

### Finding the set of representatives through bipolar fuzzy graph

**Fig. 18:**
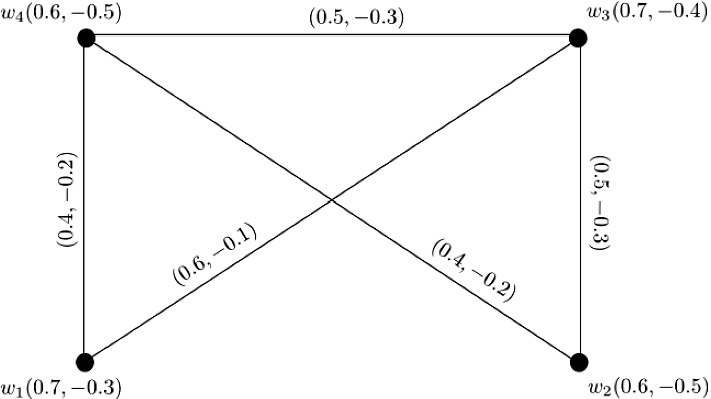
Bipolar fuzzy graph ${\mathbb {G}} = (\zeta , \sigma )$

Each student has some bad leadership qualities such as lack of enthusiasm, incompetency, poor decision-making and inflexibility. All these qualities are also uncertain in nature. Therefore, fuzziness can be added. A fuzzy graph model fails to illustrate bad leadership qualities along with good leadership qualities. A bipolar fuzzy graph model (Akram 2011) of this problem is given in Fig. 18, taking *W* as the set of vertices. The membership value of each student represents the degree of having good leadership qualities and having bad leadership qualities. For example, $$\zeta ($$Hamza$$) = (0.7, -0.3)$$ means that Hamza has $$70 \%$$ good leadership qualities and $$30 \%$$ bad leadership qualities. The edge between two students represents the degree of having common good leadership qualities and having common bad leadership qualities. For example, $$\sigma ($$Hamza, Waris$$) = (0.6, -0.2)$$ means that Hamza and Waris have $$60 \%$$ good leadership qualities in common and $$20 \%$$ bad leadership qualities in common.

The strength of connectivity between pairs of vertices of bipolar fuzzy graph ${\mathbb {G}} = (\zeta , \sigma )$ are calculated in Table 9.

**Table 9:** Strength of connectivity between pairs of vertices of ${\mathbb {G}}$

Pairs of vertices	${CONN}_{{\mathbb {G}}}{ (w, z)}$
$${ w, z }\in { W}$$	
$w_1, w_2$	$(0.5, -0.2)$
$w_1, w_3$	$(0.6, -0.2)$
$w_1, w_4$	$(0.5, -0.2)$
$w_2, w_3$	$(0.5, -0.3)$
$w_2, w_4$	$(0.5, -0.3)$
$w_3, w_4$	$(0.5, -0.3)$

**Fig. 19:**
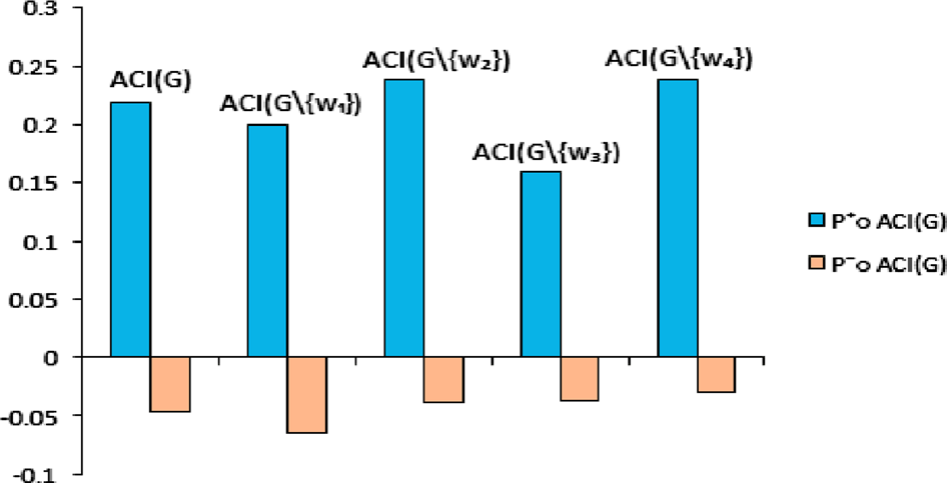
Comparison of $ACI({\mathbb {G}})$ and $ACI({\mathbb {G}} \setminus \{w_i\})$ for $i = 1, 2, 3, 4$

After calculations, we have $CI({\mathbb {G}}) = (1.314, -0.279)$ and $ACI({\mathbb {G}}) = (0.219, -0.047)$. The $CI({\mathbb {G}} \setminus \{w_i\})$ and $ACI({\mathbb {G}} \setminus \{w_i\})$ for $i = 1, 2, 3, 4$ are calculated in Table 10. The effects of deletion of different vertices on $ACI({\mathbb {G}})$ is shown in Fig. 19.

**Table 10:** $ACI({\mathbb {G}})$ after deleting a vertex from ${\mathbb {G}}$

${\mathbb {G}} \setminus \{{ w}_{ i}\}$	${ CI}({\mathbb {G}} \setminus \{{ w}_{ i}\})$	${ ACI}({\mathbb {G}} \setminus \{{ w}_{ i}\})$
${{\mathbb {G}} \setminus \{{w}_1\}}$	$(0.600, -0.195)$	$(0.200, -0.065)$
${ {\mathbb {G}} \setminus \{{w}_2\}}$	$(0.714, -0.114)$	$(0.238, -0.038)$
${ {\mathbb {G}} \setminus \{{w}_3\}}$	$(0.480, -0.110)$	$(0.160, -0.037)$
${ {\mathbb {G}} \setminus \{{w}_4\}}$	$(0.714, -0.087)$	$(0.238, -0.029)$

Therefore, $w_3$ should be the member of YDC.

### Finding the set of representatives through *m*-PF graph

**Fig. 20:**
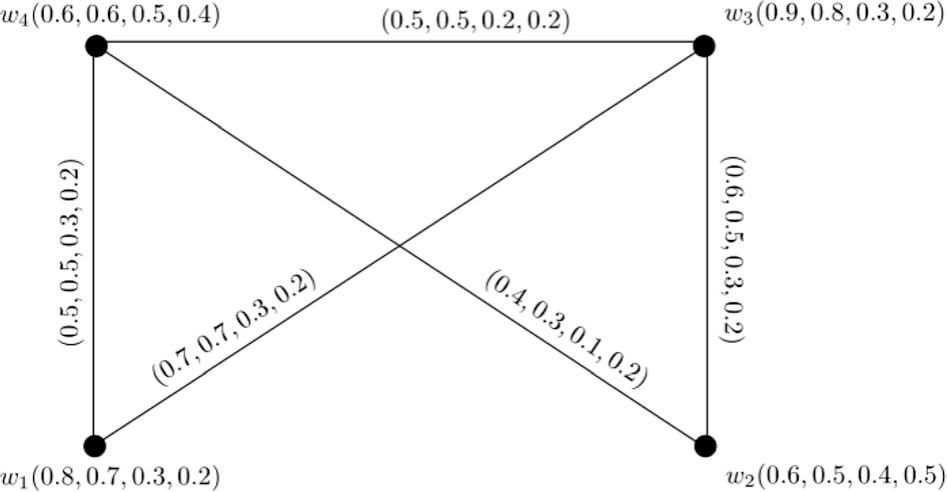
*m*-PF graph $${\mathbb {G}} = (\zeta , \sigma )$$

Note that fuzzy graph model just illustrate the overall good leadership qualities of the students. On the other hand, bipolar fuzzy graph model can illustrate the overall good leadership qualities along with the overall bad leadership qualities of the students. But, both these graph models are unable to deal with leadership qualities one by one as good leadership qualities are characterized by good communicator, approachable etc while bad leadership qualities are characterized by lack of enthusiasm, incompetency etc. Since, all these qualities are uncertain in nature. Therefore, we can associate the degree of membership value according to each single leadership quality. Fuzzy graph model and bipolar fuzzy graph model fail to handle this situation. To handle such type of problem, *m*-PF graph model is given in Fig. 20, taking *W* as the set of vertices. The membership value of each student represents the degree of communication skills, honesty, decision-making and incompetency. The description of the students can be expressed as in the following set:

$\{$Communicator, Honest, Decision-maker, Incompetent$\}$.

For example, $$\zeta ($$Hamza$$) = (0.8, 0.7, 0.3, 0.2)$$ means that Hamza is $$80 \%$$ good communicator, $$70 \%$$ honest, $$30 \%$$ good decision-maker and $$20 \%$$ incompetent. The edge between two students represents the degree of common good communication skills, honesty, good decision-making and incompetency. For example, $$\sigma ($$Hamza, Waris$$) = (0.7, 0.7, 0.3, 0.2)$$ means that Hamza and Waris have $$70 \%$$ common good communicating skills, $$70 \%$$ common honesty, $$30 \%$$ common good decision-making and $$20 \%$$ common incompetency.

The strength of connectivity between pairs of vertices of *m*-PF graph ${\mathbb {G}} = (\zeta , \sigma )$ are calculated in Table 11.

**Table 11:** Strength of connectivity between pairs of vertices of ${\mathbb {G}}$

Pairs of vertices	${CONN}_{{\mathbb {G}}}{(w, z)}$
$${ w, z }\in { W}$$	
$w_1, w_2$	(0.6, 0.5, 0.3, 0.2)
$w_1, w_3$	(0.7, 0.7, 0.3, 0.2)
$w_1, w_4$	(0.5, 0.5, 0.3, 0.2)
$w_2, w_3$	(0.6, 0.5, 0.3, 0.2)
$w_2, w_4$	(0.5, 0.5, 0.3, 0.2)
$w_3, w_4$	(0.5, 0.5, 0.3, 0.2)

**Fig. 21:**
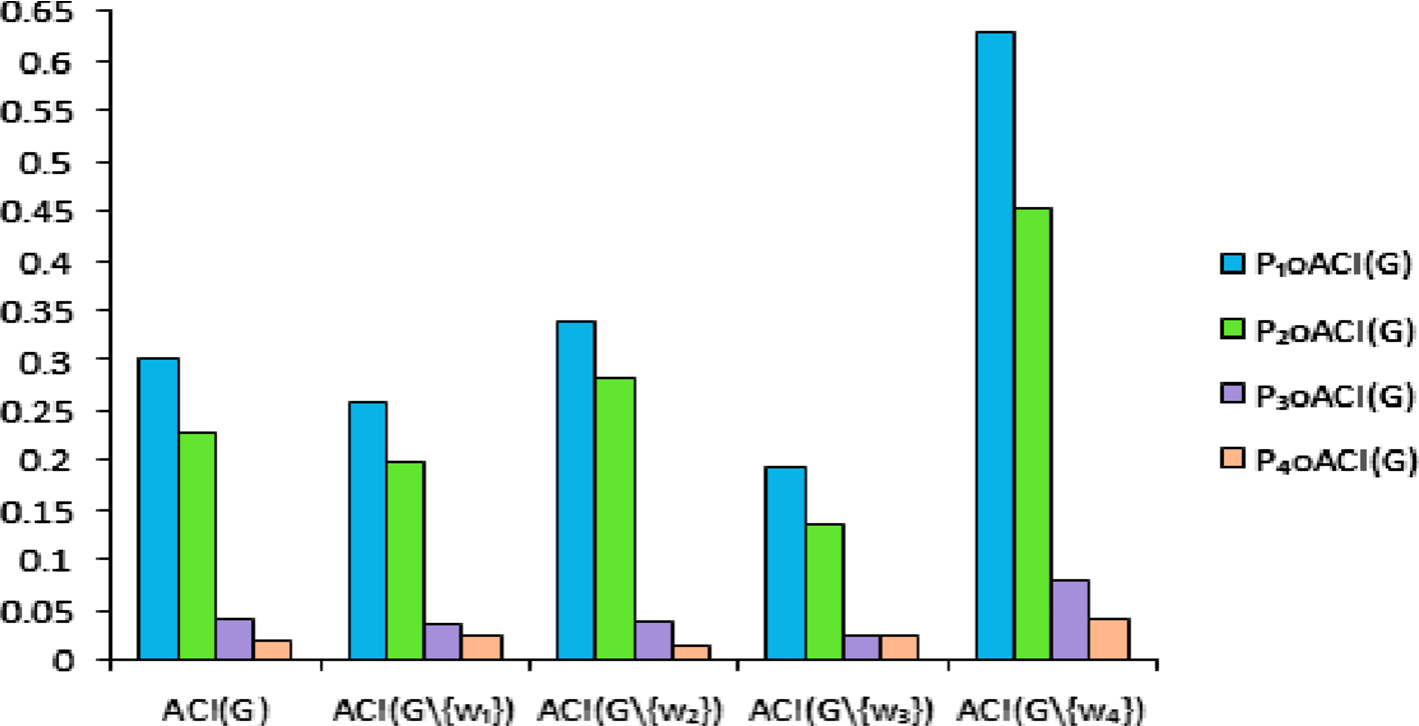
Comparison of $ACI({\mathbb {G}})$ and $ACI({\mathbb {G}} \setminus \{w_i\})$ for $i = 1, 2, 3, 4$

After calculations, we have $CI({\mathbb {G}}) = (1.806, 1.367, 0.249, 0.120)$ and $ACI({\mathbb {G}}) = (0.301, 0.228, 0.042, 0.020)$. The $CI({\mathbb {G}} \setminus \{w_i\})$ and $ACI({\mathbb {G}} \setminus \{w_i\})$ for $i = 1, 2, 3, 4$ are calculated in Table 12. The effects of deletion of different vertices on $ACI({\mathbb {G}})$ is shown in Fig. 21.

**Table 12:** $ACI({\mathbb {G}})$ after deleting a vertex from ${\mathbb {G}}$

${ {\mathbb {G}} \setminus \{{ w}_{i}\}}$	${CI}({\mathbb {G}} \setminus \{{ w}_{ i}\})$	${ACI}({\mathbb {G}} \setminus \{{w}_{ i}\})$
${ {\mathbb {G}} \setminus \{{w}_1\}}$	(0.774, 0.590, 0.106, 0.076)	(0.258, 0.197, 0.035, 0.025)
${ {\mathbb {G}} \setminus \{{w}_2\}}$	(1.014, 0.842, 0.117, 0.040)	(0.338, 0.281, 0.039, 0.013)
${ {\mathbb {G}} \setminus \{{w}_3\}}$	(0.576, 0.405, 0.077, 0.076)	(0.192, 0.135, 0.026, 0.025)
${ {\mathbb {G}} \setminus \{{w}_4\}}$	(1.890, 1.357, 0.240, 0.124)	(0.630, 0.452, 0.080, 0.041)

Therefore, both $w_1$ and $w_3$ should be the members of YDC. The feasibility and applicability of our proposed model is shown in Table 13.

**Table 13:** Comparative analysis

Model	Description	Property	Range	*ACI*(*G*)	${G }\setminus \{{ w}_{i}\}$	${ ACI}(G \setminus \{{ w}_{ i}\})$	Output
					$G \setminus \{w_1\}$	0.200	
Fuzzy graph	Describes single	Good leadership qualities	[0, 1]	0.219	$G \setminus \{w_2\}$	0.238	$w_1$ & $w_3$ should be
	property of elements				$G \setminus \{w_3\}$	0.160	the member of
					$G \setminus \{w_4\}$	0.238	YDC
					$G \setminus \{w_1\}$	$(0.200, -0.065)$	
Bipolar fuzzy graph	Describes single property	{Good leadership qualities,	$[-1, 0]\times [0,1]$	$(0.219, -0.047)$	$G \setminus \{w_2\}$	$(0.238, -0.038)$	$w_3$ should be
	with counter single	Bad leadership qualities}			$G \setminus \{w_3\}$	$(0.238, -0.038)$	the member of
	property of elements				$G \setminus \{w_4\}$	$(0.238, -0.029)$	YDC
					$G \setminus \{w_1\}$	(0.258, 0.197, 0.035, 0.025)	
*m*-PF graph	Describes more than	{Communication skills,	$[0, 1]^m$	(0.301, 0.228, 0.042, 0.020)	$G \setminus \{w_2\}$	(0.338, 0.281, 0.039, 0.013)	$w_1$ & $w_3$ should be
	one property of elements	Honesty, Decision-making	here, $m = 4$		$G \setminus \{w_3\}$	(0.192, 0.135, 0.026, 0.025)	the member of
	$(m \ge 2)$	skills, Incompetency}			$G \setminus \{w_4\}$	(0.630, 0.452, 0.080, 0.041)	YDC

## Benefits and limitations of the proposed method

The investigations above lead us to believe that the proposed connectivity analysis for *m*-PF networks may be employed efficiently to give accurate assessments of certain aspects of uncertain information. Let us summarize some of the advantages of the proposed research work:

- Connectivity analysis of *m*-PF networks enables us to apply connectivity and average connectivity indices in multi-polar uncertain real-world issues, resulting in precise solutions for more flexible graph-theoretical problems.
- The cornerstone of our model is a multi-component membership assessment. It gives us a unified expression for the adequacy of each alternative in relation with a fixed list of characteristics.
- Because various qualities of objects are captured by one proxy, more accurate information is available to perform operations conducive to well grounded decisions.

Besides these advantages, we should be aware of the limitations of proposed method.

- Although the connectivity index and average connectivity index can be used to determine the stability of an *m*-PF graph, the simultaneous comparison of numerous attributes can make it tough to achieve fully convincing selections.
- The proposed model delivers flexible outcomes, however it is difficult to manage multi-components of membership values in the case of large datasets, as they impose a computational burden and increase complexity.

## Conclusion and future directions

One of the key factors affecting a network is connectivity. In this article, two distinct network parameters, namely, the connectivity index and average connectivity index, are described for *m*-PF graphs. These indices permit to evaluate the stability of a *m*-PF network. Characterizations for various kinds of *m*-PF graphs are obtained. Specifically, the effects of deleting a vertex or an edge in *m*-PF graphs, and the bounds in the case of specific *m*-PF graphs, are analyzed. Algorithms related to these concepts are given. The results that have been obtained can be helpful in the quantitative inspection of human trafficking, Internet routing, etc. In order to demonstrate and validate the proposed algorithm, our work has provided useful tools for a more efficient application of *m*-PF graphs in practice. We have applied them in a particular product manufacturing problem, and explained it with reference to a general algorithm designed to easily understand the method. Finally, we have provided a comparative analysis to prove the feasibility and validity of proposed method. Thus, our approach may offer flexible solutions to several graph-theoretical multi-polar uncertain real-world scenarios by managing multi-components of the membership values for large datasets.

This research work can be further extended to include the analysis of (1) Operators and algorithms to efficiently handle multi-polarity; (2) Connectivity indices of *m*-PF rough graphs; (3) Cyclic connectivity index of *m*-PF graphs; (4) Average cyclic connectivity index of *m*-PF graphs; (5) Wiener index of *m*-PF graphs.

## Delarations

### Data availability

No data were used to support this study.
